# Identification of Secondary Metabolites from the Mangrove-Endophyte *Lasiodiplodia iranensis* F0619 by UPLC-ESI-MS/MS

**DOI:** 10.3390/metabo13080912

**Published:** 2023-08-03

**Authors:** Lizbeth M. Delgado Gómez, Daniel Torres-Mendoza, Kathleen Hernández-Torres, Humberto E. Ortega, Luis Cubilla-Rios

**Affiliations:** 1 Laboratorio de Bioorgánica Tropical, Facultad de Ciencias Naturales, Exactas y Tecnología, Universidad de Panamá, Panamá 0824, Panama; 13liz2014@gmail.com (L.M.D.G.); daniel-t.torres-m@up.ac.pa (D.T.-M.); kathleen-j.hernandez-t@up.ac.pa (K.H.-T.); humberto.ortegad@up.ac.pa (H.E.O.); 2 Departamento de Química Orgánica, Facultad de Ciencias Naturales, Exactas y Tecnología, Universidad de Panamá, Panamá 0824, Panama; 3 Vicerrectoría de Investigación y Postgrado, Universidad de Panamá, Panamá 0824, Panama; 4 Programa de Maestría en Microbiología Ambiental, Vicerrectoría de Investigación y Postgrado, Universidad de Panamá, Panamá 0824, Panama

**Keywords:** *Lasiodiplodia iranensis*, jasmonates, mangrove-endophytes, MS/MS fragmentation analysis

## Abstract

*Lasiodiplodia* is a widely distributed fungal genus, frequently found in tropical and subtropical regions where it can cause disease in important crops. It represents a promising source of active secondary metabolites with uses in chemical, pharmaceutical, and agrochemical processes. In this study, the strain *Lasiodiplodia iranensis* F0619 was isolated from the mangrove *Avicennia ger-minans*, collected from Sarigua National Park in the Republic of Panama. Fractions of crude extract were analyzed by UPLC-ESI-MS/MS, and five compounds, previously reported from *Lasiodiplodia* genus were identified, including 11,12-didehydro-7-*iso*-jasmonic acid (1), 4,5-didehydro-7-*iso*-jasmonic acid (2), *cyclo*-(L-Leu-L-Pro) (3), jasmonate-threonine (4), and abscisic acid (5). We describe and analyze their MS/MS fragmentation patterns to confirm the compounds ‘chemical structures.

## 1. Introduction

Endophytes are microbes that live inside the tissues of a host organism without causing harm or disease. They have gained increasing attention in recent years due to their ability to boost host growth and yield, strengthening responses to biotic and abiotic stress in multi-factorial, complex ways unique to each microbe-host combination [1]. Endophytic fungi have the potential to produce novel chemical compounds with varied biological activities and high relevance in medicinal, pharmaceutical, and agricultural applications [2,3,4,5].

Mangrove ecosystems represent a hotspot of biodiversity whose flora, fauna, and microbiota remain largely unexplored. In particular, mangrove-associated fungi are a rich source of new bioactive compounds with unique chemical scaffolds and have gained a growing interest in biotechnology and pharmacology [6,7,8,9,10].

Members of the fungal genus *Lasiodiplodia* (Family: Botryosphaeriaceae), are endo-phytes, saprobes, or destructive plant pathogens. They have produced a huge structural diversity of secondary metabolites that display a wide range of biological activities such as antibiotic, antiproliferative, antioxidant, anti-inflammatory, antiparasitic, phytotoxic, neuroprotective, cytotoxic and xanthine oxidase, quorum sensing, and α-glucosidase in-hibition among others [11,12,13,14,15,16,17]. Mangrove-endophytic *Lasiodiplodia* have been reported to produce β-resorcylic acid derivatives and preussomerins [18,19,20,21,22]. Among *Lasiodiplodia* secondary metabolites, jasmonates, represents an important group of compounds derived from α-linolenic acid. Jasmonates have a cyclopentanone ring as their main core to which one aliphatic and one carboxylic side chain is attached. They have biological activity as phytohormones and key mediators on plant responses to insects, necrotrophic pathogens, and environmental stresses. As such they have potential applications as agricultural, cosmetic, pharmaceutical, and nutraceutical molecules [23,24,25,26,27].

Ultra-performance liquid chromatography coupled to electrospray ionization tandem mass spectrometry (UPLC-ESI-MS/MS) is a separation-base technique suitable for the analysis of complex mixtures that typically pose isolation and dereplication challenges in natural product research. This technique increases the resolution of chromatographic peaks and provides a precise characterization of each metabolite and a fragmentation pattern to aid structural characterization and identification [28,29,30]. In this work, we describe the structural identification of compounds present in the extract of mangrove-endophyte *L. iranensis* F0619.

## 2. Material and Methods

### 2.1. Chemicals and Reagents

All the following chemicals were acquired from Sigma-Aldrich^®^ (Sigma-Aldrich, St. Louis, MO, USA): glycerol, dimethyl sulfoxide (DMSO). Ethyl acetate, acetone, dichloromethane, acetonitrile, and methanol used for extraction, and thin layer (TLC) and column chromatography were American Chemical Society grade (Tedia^®^, Tedia Company Inc., Fairfield, OH, USA). Hexane, isopropanol, and ethyl acetate for high-performance liquid chromatography (HPLC) were HPLC grade (Tedia^®^, Tedia Company Inc., or Merck, EMD Millipore Corporation, Burlington, MA, USA). The acetonitrile (AcCN) for the liquid chromatography-mass spectrometry (LC-MS) analysis was LC-MS grade (HPLC/Spectro, Tedia^®^, Tedia Company Inc., Fairfield, OH, USA).

### 2.2. Strain Isolation and Identification

A healthy specimen of *Avicennia germinans* (Family: Acanthaceae) was collected in Sarigua National Park, Province of Herrera, Republic of Panama in September 2004. Mature leaves were surface sterilized following our protocol previously reported in [31]. Isolate F0619 was successively re-plated until a pure strain was obtained. The pure fungal strain was stored at −80 °C in a solution of 10% glycerol and preserved until further use. The strain was kept in the culture collection of the University of Panama under the code [369B3-17(198)].

Before the reactivation of the strain, a culture recovery protocol was applied [32]. Mycelial plugs (5 mm) were aseptically transferred into Petri dishes containing malt extract-agar (MEA) and incubated at 26 °C for 7 to 15 days, then storage under sterile distilled water.

The morphological identification was conducted in MEA cultures. Macroscopic characteristics of the culture were observed for 15 days at 26 °C under permanent light. The fungal structures were imaged under an Leica DM200 LED microscope with photographic camera MC170 HD. To examine details, low-distortion tape touch and microculture methods were employed [33]. For microcultures, glass Petri dishes with sterile and water-saturated absorbent paper were used. The samples were placed on 14 mm agar plugs and covered with sterile coverslips. After incubating for 5–7 days, the samples were transferred onto a microscope slide with a drop of lactoglycerol blue stain on sterile water.

1. Active mycelium from three plugs was aseptically transferred to two Petri dishes (145 mm × 20 mm) of fresh MEA and incubated at 26 °C for 10 days. Subsequently, the mycelia were ground with liquid nitrogen until a fine powder was obtained, then was stored at −80 °C until further analysis. Genomic DNA (gDNA) was extracted from 125 mg of frozen biomass using the *Quick*-DNA Fungal/Bacterial Miniprep Kit (Zymo Research Corp., Irvine, CA, USA, Cat No. D6005) protocol according to the manufacturer’s instructions. The quality and concentration of gDNA were quantified using a Nanodrop^TM^ Spectrophotometer (ThermoFisher Scientific, Waltham, MA, USA).

Molecular identification was carried out by analysis of fungal DNA sequences of the conserved complete internal transcribed region (ITS 1, 5.8S, ITS2). The extracted gDNA was amplified by the final polymerase chain reaction (PCR) using the ITS universal oligonucleotides 1 (5′-TCCGTAGGTGAACCTGCGG-3′), ITS 4 (5′-TCCTCCGCTTATTGATATGC-3′), and ITS 5 (5′-GGAAGTAAAAGTCGTAACAAGG-3′) [34,35].

PCR amplification was performed using Taq DNA polymerase 5 U/μL (Invitrogen; USA). The PCR mix contained 2.5 μL of 10X PCR buffer, 0.5 μL of dNTPs 10 mM, 1.25 μL of 250 mM MgCl_2_, 0.6 μL for each primer ITS1 and ITS4, 0.125 μL of Taq DNA polymerase 5U/μL, 0.2 μL DMSO, 15.2 μL of sterile ultrapure water, and 4.0 μL of the fungal gDNA. The final reaction mixture volume was 25 μL with the following cycle profile: an initial denaturation cycle at 94 °C for 3 min, followed by 30 cycles at 94 °C for 45 s, annealing stage at 52 °C for 30 s, elongation at 72 °C for 50 s, and a final extension at 72 °C for 10 min.

The PCR products were verified by 0.9% agarose gel electrophoresis at 60 V, to confirm the fragment’s size, which was determined by comparison with the 100 bp/amresco marker (VWR Life Science^®^; Radnor, PA, USA). PCR amplicons were sent to Psomagen (Psomagen Inc., Rockville, MD, USA) for sequencing. ITS raw sequences were assembled and edited using BioEdit^®^ Sequences Alignment Editor (version 7.1) [36,37], and the edited nucleotide sequences were compared with sequences from the GenBank^®^ using the Basic Local Alignment Search Tool (BLAST^®^) [38]. The edition of alignments was performed manually to exclude incomplete sections at the end of sequences before analysis.

Taxonomic identification was assigned by sequence similarity to public data in NCBI GenBank^®^ and morphological data. The sequence obtained was deposited in the GenBank^®^ database with their accession number OP684132. A phylogeny was estimated with sequences available in public data from NCBI GenBank^®^.

### 2.3. Initial Cultivation and Extraction

The fungal strain was inoculated in Petri dishes with MEA and grown in an incubation chamber at 26 °C with permanent light. After 15 days, media with mycelia were cut into pieces with a sterile spatula and stored in sterile plastic bags at −20 °C.

The frozen sample was placed in a 1 L beaker with 200 mL of ethyl acetate for 20 min. Subsequently, the sample was ground using a Polytron homogenizer. To avoid excessive heat production, this operation was carried out in an ice bath. The homogenized sample was then filtered using filter paper under vacuum. The filtrate was placed in a separatory funnel and the organic phase was obtained, then the aqueous phase was extracted with ethyl acetate (2 × 100 mL). The organic phases were combined and placed in a flask to evaporate the solvent under reduced pressure. The extract was re-suspended in methanol and transferred to amber glass vials. Finally, the MeOH was evaporated, and the vial was weighed to determine the mass of the extract. 

### 2.4. Fractionation of the Crude Extract and Analysis of Fractions by HPLC

The crude extract was fractionated using column chromatography on silica gel 100 (particle size 0.063–0.200 mm, 70–230 mesh ASTM; EM Science, Gibbstown, NJ, USA) by eluting in a stepwise gradient of 250 mL dichloromethane (CH_2_Cl_2_)/Acetone/acetonitrile (AcCN) (100:0:0; 60:40:0; 40:60:0; 0:100:0, 0:0:100). The fractions were analyzed and combined based on their profile on Merck TLC sheets (silica gel 60 F_254_, aluminum-supported, layer-thickness 200 μm).

HPLC analysis was carried out on a Waters Delta 600 LC system, including a Waters 600 pump and a Waters 996 photodiode array detector, and a normal phase silica gel column (YMC-Pack SIL, 5 μm, 10 × 150 mm, YMC America, Inc., Devens, MA, USA) using different mixture gradients from 50-40 % hexane or isopropanol—ethyl acetate during 5 minutes and then to 100% ethyl acetate in 60–65 min at 1.5 mL/min.

### 2.5. Analysis of Fractions by LC-PDA-MS/MS

Fractions from the organic extract were diluted in methanol suitable for LC-MS at 1.0 mg/mL, filtered through a 0.22 μm polytetrafluoroethylene (PTFE) membrane, and analyzed in a Waters ACQUITY class-H UPLC^TM^ system using an Acquity UPLC^®^ BEH (1.7 μm) C18, reverse phase LC column, 2.1 × 100 mm, a Waters PDA eλ Detector, and a XEVO^®^ TQD spectrometer (Waters Corporation, Milford, MA, USA) supplied with an electrospray ionization (ESI) source. The gradient for the chromatographic separation was carried using a step UPLC run of acetonitrile and water (H_2_O), starting at 10:90% AcCN-H_2_O to 100% AcCN in 10 min and returning to initial condition of 10:90% AcCN in 2 min at a flow rate of 0.2 mL/min throughout the run. The ESI-MS/MS parameters were set as follows: capillary voltage 3.5 kV; cone voltage 20 V; source temperature 150 °C; desolvation temperature 450 °C; desolvation gas flow 600 L/h. Mass spectrometry data were acquired in positive ion mode with a detection range of 175–2000 *m*/*z* using the MS Survey Scan method for MS/MS fragmentation. MassLynx^TM^ software (version 4.2) was used for data acquisition and processing.

## 3. Results

### 3.1. Characterization and Identification of Biological Material

**Figure 1:**
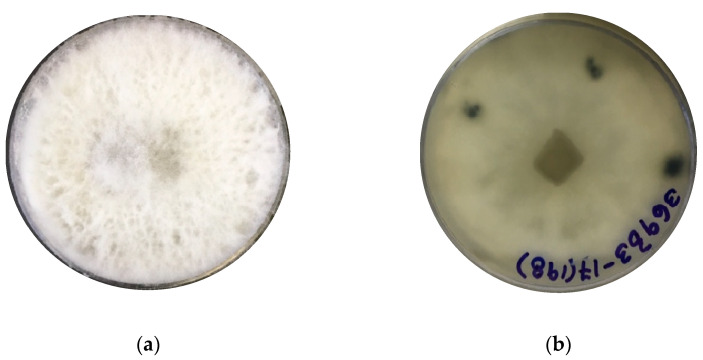

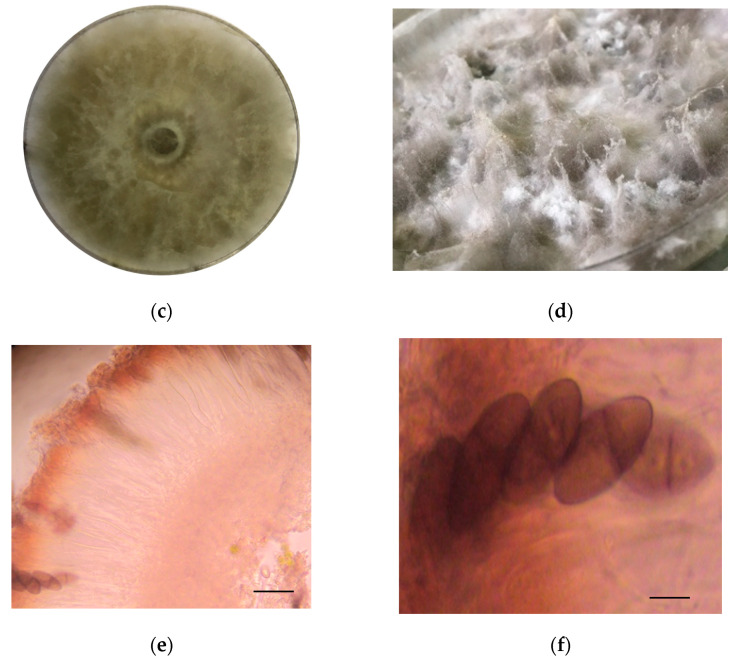
Lasiodiplodia iranensis F0619. (**a**) Colony morphology, front side at 7 days of cultivation; (**b**) Colony morphology reverse side at 7 days of cultivation; (**c**,**d**) Culture on mal extract-agar after 15 days of cultivation; (**e**) Hyaline paraphysis on MEA; (**f**) Dark mature conidia. ― scale bar in (**e**,**f**) = 10 μm.

The identification of strain F0619 on malt extract-agar was based on the morphology and phylogenetic analysis. Incubation was performed at 26 °C for 7–15 days, upon which the strain formed colonies on MEA displaying characteristic hyphal structures [39,40,41]. The colonies on MEA reach a diameter of 40 mm, reaching the lid of the Petri dish after seven days of growth under light at 26 °C (Figure 1a,b). After ten days of cultivation, the strain developed a fluffy white aerial mycelium then changed to dark gray at the end of the cultivation phase (Figure 1c,d). Hyaline paraphyses and dark brown septate conidia were observed (Figure 1e,f). There was an absence of sexual morphology and conidiophores.

The ITS region of the ribosomal DNA was amplified by PCR and sequenced. Better amplification was obtained using the ITS 5–ITS 4 primers (Figure S1). The alignments were manually refined when necessary to exclude incomplete portions at the end of the sequence before analyses. Maximum Likelihood analyses were performed with MEGA11 [42] and the heuristic search was obtained automatically by applying the Maximum Parsimony method. A discrete Gamma distribution was used to model evolutionary rate differences among sites [5 categories (+G, parameter = 0.3993)]. The evolutionary history was inferred by using the Maximum Likelihood method and the Hasegawa-Kishino-Yano model [43]. Parameters for maximum likelihood were set to rapid bootstrapping and the analysis was carried out using 1000 replicates. The phylogenetic tree constructed from the ITS gene PCR amplification indicated that the strain F0619 belonged to the genus *Lasiodiplodia* with the highest similarity to *L. iranensis* RB31 (99.65%, accession number: KY052971).

### 3.2. Extraction, Fractionation, and TLC and HPLC Profile of Fractions

The fungal strain *L. iranensis* F0619 was cultured in 94 Petri dishes containing MEA in an incubation chamber at 26 °C during 15 days with permanent light and then extracted with ethyl acetate to obtain 1.535 g of the organic crude extract. 

An aliquot of 1.0 g of the crude extract was fractionated using silica gel column chromatography eluting in a stepwise gradient of CH_2_Cl_2_-acetone-AcCN to yield 5 fractions according to TLC profiles (A–E) (fraction A = 32.8 mg; fraction B = 318.8 mg; fraction C = 131.3 mg; fraction D = 84.8 mg; and fraction E = 25.1 mg).

The fractions were analyzed by normal phase silica gel TLC and HPLC resulting in very complex profiles of compounds (Figure S2). Despite this fact, fraction C (131.3 mg) presented a less complex profile coming from a medium polarity eluent (40:60:0 CH_2_Cl_2_-acetone-AcCN) making it suitable for UPLC-MS/MS analysis. Compared to those in HPLC, the UPLC-DAD chromatograms showed the best possible separation of some peaks letting the identification of some compounds by tandem mass spectrometry (Figure S3).

### 3.3. Metabolites Identification by Liquid Chromatography-Tandem Mass Spectrometry

A literature search allows us to recognize that in fraction C were present molecular ions that matched with the corresponding to five compounds already reported for the genus Lasiodiplodia, including jasmonates, a diketopiperazine, and a sesquiterpenoid (Table 1). Applying logical fragmentation mechanisms lets us justify the MS-MS data obtained for each of the five compounds present in the extract. The formation of some of the characteristic fragments for each of the structures of the proposed compounds is explained below.

## 4. Discussion

### 4.1. Fungal Strain Identification

**Figure 2:**
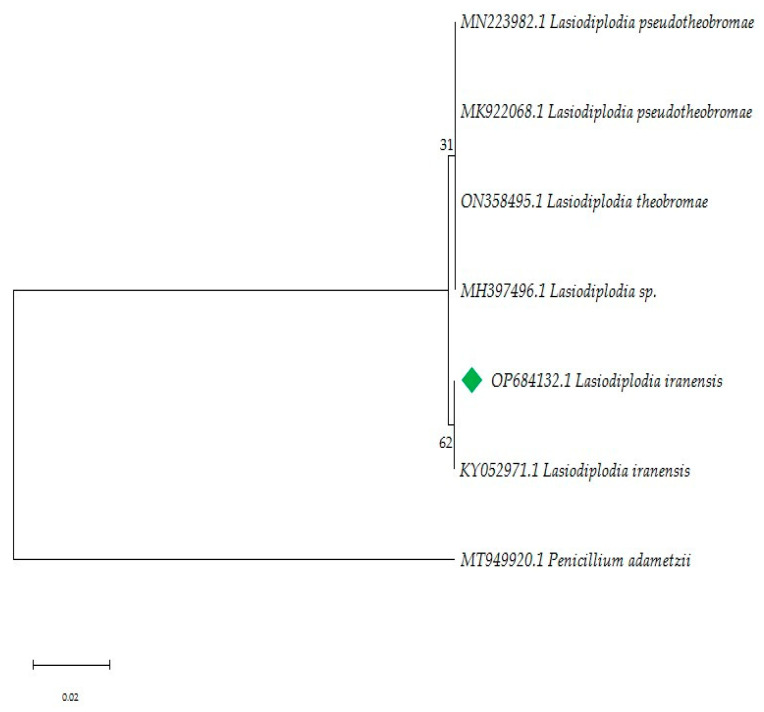
Phylogenetic analysis of the ITS region based on the Maximum Likelihood method. The tree was rooted with *Penicillium adametzii* (accession number MT949920.1) In green, focal strain *L. iranensis* F0619. There is, next to the branches, the percentage which associated taxa clustered together. The highest log-likelihood values were −1596.61.

Historically, studies on Lasiodiplodia have focused on pathogenic species, likely due to the agroeconomic impact of the fungus. Additionally, the high morphological variability among species makes it difficult to reliably distinguish differences between *Lasiodiplodia* species [39,46]. Macro and micromorphology identification were performed to support the phylogenetic reconstruction. A total of 7 similar sequences were obtained from GenBank^®^, each with similarities over 98% to the focal strain. Sequences were grouped into two clades: clade 1, included *Lasiodiplodia* sp., *L. theobromae* and *L. pseudoiranensis* (Bootstrap 31%), while clade 2 consisted of *L. iranensis* (Bootstrap 62%, Figure 2). Morphological and phylogenetic studies are essential for a broader identification and provide support for understanding fungal function in symbiosis with other species.

### 4.2. Structural Characterization of Metabolites

**Figure 3:**
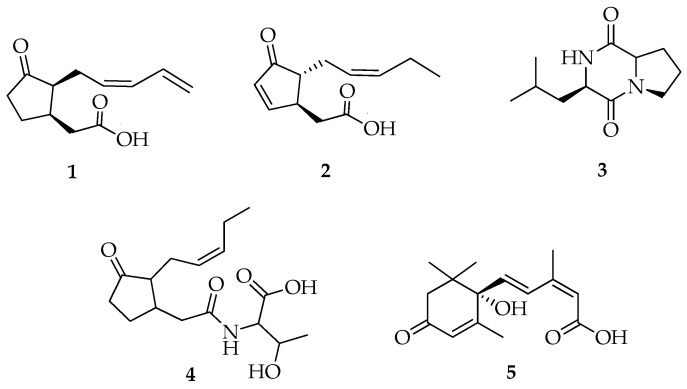
Structure of compounds **1**–**5**.

Compounds **1**, **2,** and **4** (Figure 3) had ESI-MS/MS spectra (Figures S4–S7, S10, and S11) containing protonated molecules at *m*/*z* 209.1, 209.3, and 312.2 [M + H]^+^ that matched with the molecular mass for the previously reported jasmonates 11,12-didehydro-7-*iso*-jasmonic acid, 4,5-didehydro-7-*iso*-jasmonic acid, and jasmonate-threonine, respectively, from *L. theobromae* [11]. 

**Scheme 1:**
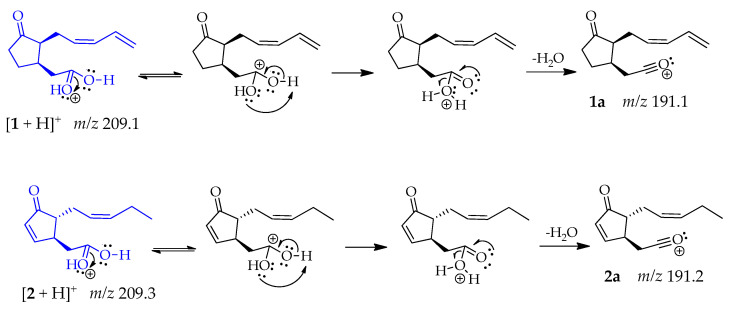
Generation of fragment at *m*/*z* 191 in compounds **1** and **2**.

Compounds **1** and **2** produced the same fragment at *m/z* 191 [M + H − 18]^+^ that was consistent with a loss of water, caused by the protonation of the oxygen from the carboxylic acid carbonyl group, which has higher electron density. This oxygen is highly electronegative but can share its electrons and allow a double resonance that results in the release of a water molecule, leading fragments **1a** and **2a**, respectively [47] (Scheme 1).

**Scheme 2:**
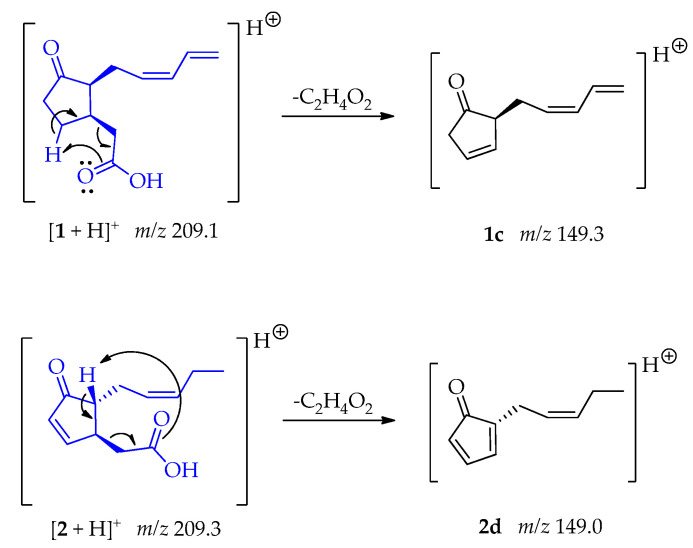
Generation of fragment at *m/z* 149 from compounds **1** and **2**.

The fragments **1c** and **2d** at *m*/*z* 149 [M + H – C_2_H_4_O_2_]^+^ were generated due to a McLafertty rearrangement involving the migration of γ-hydrogens to the carboxyl group, leading to the loss of ethanoic acid [48]. In compound **2** the formation of the conjugate diene could enable the generation of **2d** (Scheme 2).

**Scheme 3:**
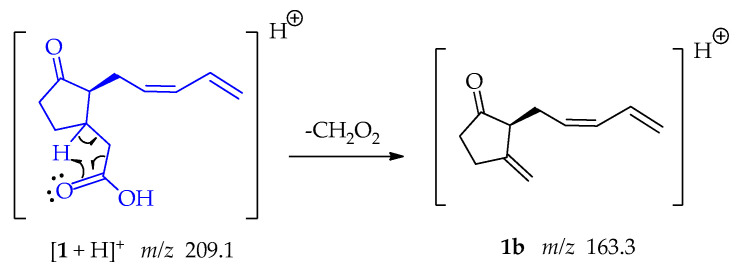
Generation of fragment at *m*/*z* 163 from compounds **1**.

Fragment **1b** at *m*/*z* 163.3 was formed when the oxygen captures a proton from position β to the carbonyl group, the electrons that form the bond with this hydrogen resonate, allowing the loss of CH_2_O_2_ as shown in Scheme 3.

**Scheme 4:**
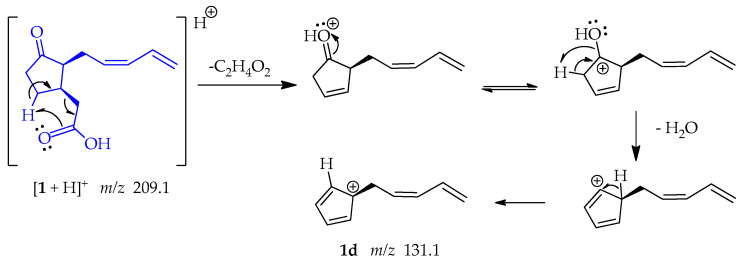
Generation of fragment at *m*/*z* 131.1 from compound **1**.

The shifting of a γ-hydrogen following a retro-heteroene reaction, specifically a McLafferty-type rearrangement in **1**. This reaction involves the transfer of the hydrogen to the carbonyl-bound oxygen through a 6-membered transition state, resulting in the production of an ene fragment. Additionally, this reaction facilitates the loss of ethanoic acid (C_2_H_4_O_2_). Thereafter, the positive charge is placed on the oxygen of the carbonyl group, and the electrons resonate toward the oxygen, forming an OH group. This OH group facilitates the capture of an adjacent proton and the subsequent loss of water. This sequence of reactions yields a resonance-stabilized tertiary allylic carbocation **1d** at *m*/*z* 131.1 (Scheme 4) [49].

**Scheme 5:**
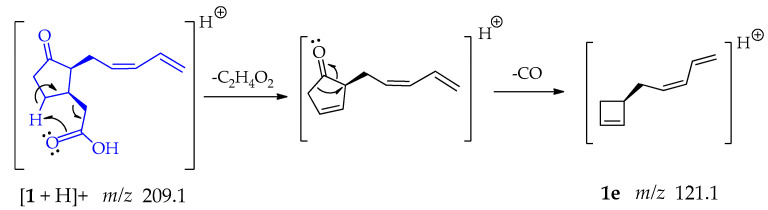
Generation of fragment at *m*/*z* 121.1 from compound **1**.

The formation of fragment **1e** at *m*/*z* 121.1 (Scheme 5) initially occurs with the above explained McLafferty-type rearrangement guided to the loss of ethanoic acid; followed by detachment of CO from the cyclopentanone ring, which is a relatively common mechanism in cyclic carbonyl compounds that occurs in a single step [48,50].

**Scheme 6:**
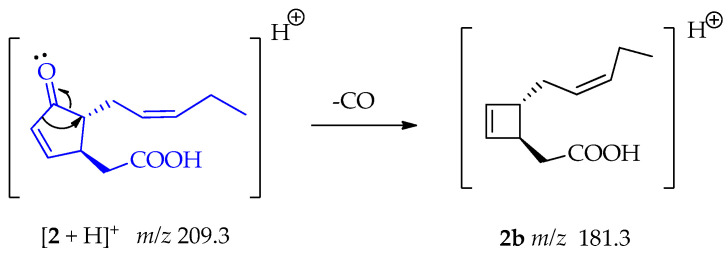
Generation of fragment at m/z 181.3 from compound **2**.

The fragment at *m*/*z* 181.3 (**2b**) indicates a similar loss of CO from compound **2**, before the subsequent loss of the C_2_H_4_O_2_ fragment, as shown in Scheme 6 [50].

**Scheme 7:**
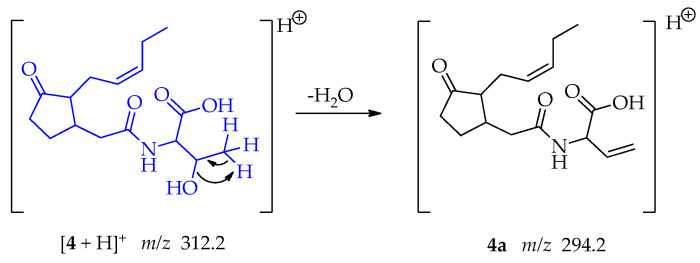
Generation of fragment at *m*/*z* 294.2 from compound **4**.

Compound **4,** a jasmonate derivative exhibited a different MS/MS fragmentation pattern than observed in compounds **1** and **2**. The main fragment observed corresponded to the loss of water from the threonine moiety; involving an intramolecular 1,2-elimination and the formation of a new π bond in the fragment **4a** at *m*/*z* 294.2 (Scheme 7) [50].

**Scheme 8:**
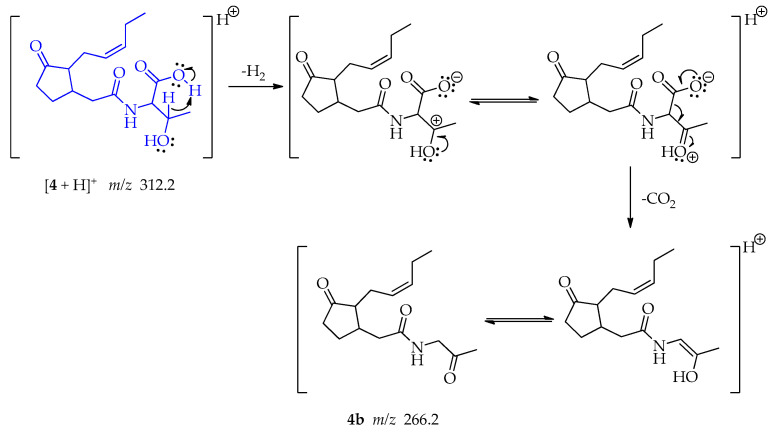
Generation of fragment at *m*/*z* 266.2 from compound **4**.

A far-charge reaction occurs in the threonine, implying a 6-membered transition state that allows the concerted elimination of H_2_ and CO_2_ leading the formation of fragment **4b** at *m*/*z* 266.2 whose keto form is more stable (Scheme 8) [51]. 

The joint double elimination may occur when the hydrogen at the β-position of the carboxyl group acts as a base and interact with the acidic proton allowing the formation of H_2_ and generating a carbocation. This could be followed by the formation of a double bond with the hydroxy group. At the same time, the oxo anion, poised by the loss of the proton, resonates, and enables the loss of CO_2_ and the formation of a secondary carbanion that forms a double bond with the carbocation, producing an enol. A keto-enol tautomerism may be produced.

**Scheme 9:**
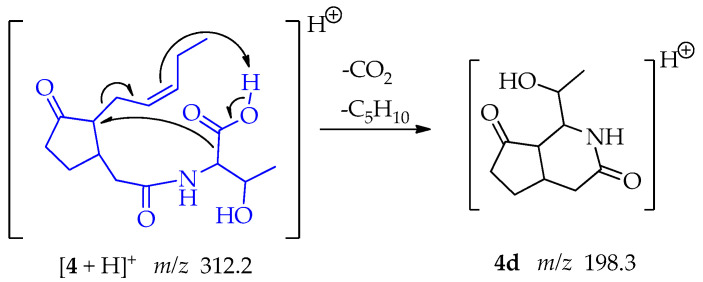
Generation of fragment at *m*/*z* 198.3 from compound **4**.

In the case of the fragment at *m*/*z* 198.3, the pi electrons of the pentenyl side chain act as a base and capture the proton of the threonine carboxyl group. Two eliminations reactions follow: the oxo anion, poised by the loss of the proton, resonates, and enables the loss of CO_2_, producing at the same time a secondary carbanion. The secondary carbocation formed by the movement of the double bond is compensated by resonance allowing the loss of the C_5_H_10_ chain leaving a carbocation in C-7 located in the cyclopentanone ring. The carbanion binds the carbocation forming fragment **4d** at *m*/*z* 198.3, a 6-member ring lactam attached to the cyclopentanone ring (Scheme 9).

For a summarized diagram of all principal fragments of compounds **1**, **2** and **4** see Figures S2, S4, and S8. 

**Scheme 10:**
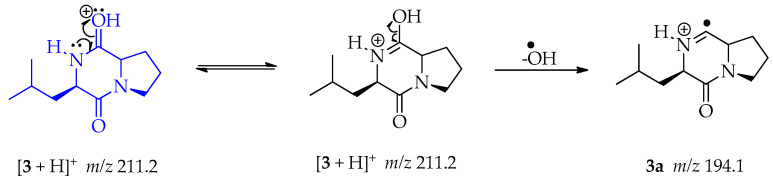
Generation of fragment at m/z 194.1 from compound **3**.

The ESI-MS/MS data collected for compound **3** showed a protonated molecule ion [M + H]^+^ at *m*/*z* 211.2 (Figures S8 and S9). This mass was consistent with the 2,5-diketopiperazine cyclo-(L-Leu-L-Pro) and the fragmentation pattern was coherent with the previous report [52]. The loss of CO directly from the protonated molecule is a common fragmentation mechanism characteristic of the analyzed diketopiperazines. We propose a pathway to fragment at *m*/*z* 194.1 not presented in the above-mentioned publication. During the ionization processes the proton is located on the oxygen, followed by double resonance producing **3**. Then a homolytic cleavage permits the loss of the hydroxy radical and lets the formation of fragment **3a** at *m*/*z* 194.1 (Scheme 10) [53].

**Scheme 11:**
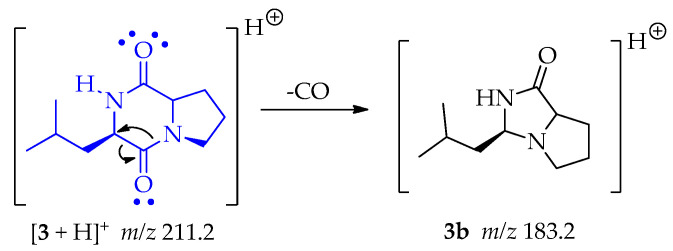
Generation of fragment *m*/*z* 183.2 from compound **3**.

There is a classic rearrangement of carbonyl compounds that lets to the loss of carbon monoxide (CO) and generating the fragment **3b** at *m*/*z* 183.2 as shown in Scheme 11 [50].

**Scheme 12:**
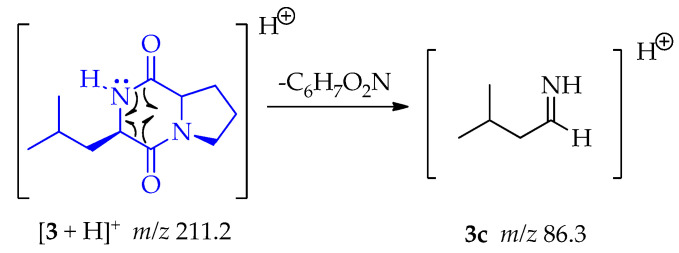
Generation of fragment at *m*/*z* 86.3 from compound **3**.

A homolytic rupture at α-positions of both carbonyl groups, as is indicating in 3, let the formation of **3c** (*m*/*z* 86.3) by the loss of C_6_H_7_O_2_N as shown in Scheme 12.

**Scheme 13:**
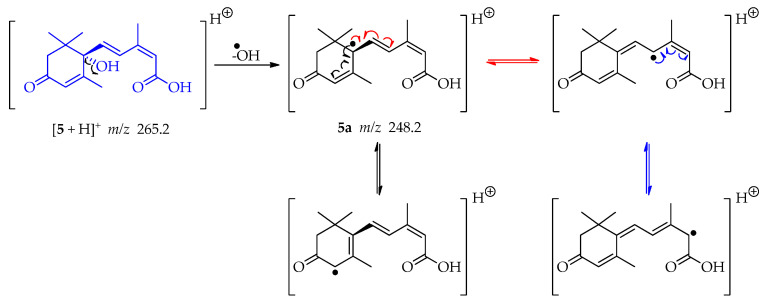
Generation of fragment at *m*/*z* 248.2 from compound **5**.

The ESI-MS/MS data collected for compound **5** showed a protonated molecule ion [M + H]^+^ at *m*/*z* 265.2 (Figures S12 and S13). This mass was consistent with the sesquiterpene abscisic acid. By elimination of the hydroxy radical of the tertiary alcohol in compound **5**, a tertiary radical is generated **5a** (*m*/*z* 248.2). It is a double allylic radical and possesses several resonance structures (Scheme 13) having therefore a reasonable stability. The loss of water, for this tertiary alcohol, cannot occur since C-2′ and C-6′ are quaternary and C-5 could form an unstable allene [53].

**Scheme 14:**
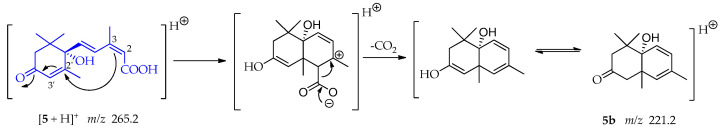
Generation of fragment at *m*/*z* 221.2 from compound **5**.

A Michael addition follows by the loss of CO_2_ is consistent with fragment **5b** at *m*/*z* 221.2 in its keto form as shown in Scheme 14 [54]. In this case, the double bond between C-2 and C-3 is the nucleophile and the double bond between C-2′-C-3′ is the Michael acceptor. This addition leads to a cyclization and an electron rearrangement allows the loss of CO_2_. 

**Scheme 15:**
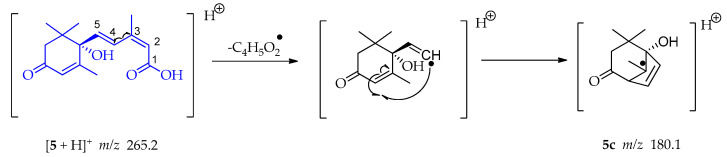
Generation of fragment at *m*/*z* 180.1 from compound **5**.

A homolytic rupture between C-3 and C-4 occurs in compound **5**, followed by an electronic structure arrangement leading to a cyclization and a bicyclic tertiary radical **5c** at *m*/*z* 180.1 (Scheme 15) [55].

In this study, we used UPLC-MS/MS to identify five secondary metabolites produced by the genus *Lasiodiplodia* present in the organic extract obtained from the culture of *L. iranensis* F0619 in MEA, including the generation of characteristic fragments and plausible mechanisms of fragmentation.

Jasmonates (including compounds **1**, **2,** and **4**) and abscisic acid (**5**) have been considered essentially secondary metabolites for plant survival in nature. Jasmonates and analogs can act as regulators of plant responses to environmental and biotic stress, such as ozone exposure, wounding, water deficit, UV light, pathogen, insects, and pest attack. They also can be involved in plant developmental processes including root growth, production of viable pollen, seed germination, tuberization, fruit ripening, tendril coiling, leaf abscission, and senescence [56,57]. Endophytic fungi are capable to produce important phytochemical compounds that were initially thought to be produced only by host plants, therefore they can be considered as an alternative source for bioactive natural products.

## Figures and Tables

**Table 1:** Identification of secondary metabolites in *L. iranensis* F0619.

Compound	Retention Time in Minutes (*t*_R_)	UV (nm)	Molecular Weight (g/mol)	[M + H]^+^ (*m*/*z*)	MS/MS Fragments Ions	Reference
**1**	1.60–1.73	195, 233	208.26	209.1	191.1, 163.3, 149.3, 131.1, 121.1, 107.1, 71.0	[11]
**2**	3.55–3.95	227	208.25	209.3	191.2, 181.3, 167.3, 153.2, 149.0, 131.1, 121.0, 107.0, 93.1	[11]
**3**	4.30–4.50	205	210.27	211.2	194.1, 183.2, 166.3, 137.8, 114.3, 98.0, 86.3, 69.9	[11,44]
**4**	5.36–5.74	208, 235, 275	311.37	312.2	294, 266, 248, 198, 181, 125, 86	[11,24,45]
**5**	5.84–5.98	243	264.32	265.2	248.2, 221.2, 205.1, 180.1	[11]

## Data Availability

The DNA sequences are deposited in GenBank^®^ (https://ncbi.nlm.nih.gov/genbank (accessed on 24 June 2023)). The data presented in this study are available in the main article and the supplementary material.

## Supplementary Materials

The following supporting information can be downloaded at: https://www.mdpi.com/article/10.3390/metabo13080912/s1, Figure S1: Quality of PCR products checked on agarose electrophoresis gel; Figure S2: TLC and HPLC analysis of fractions of *L. iranensis* F0619; Figure S3: UPLC-DAD chromatogram from fraction C; Figure S4: ESI^+^—MS/MS spectra of compound **1**; Figure S5: Fragmentation pattern for compound **1**; Figure S6: ESI^+^—MS/MS spectra of compound **2**; Figure S7: Fragmentation pattern for compound **2**; Figure S8: ESI^+^—MS/MS spectra of compound **3**; Figure S9: Fragmentation pattern for compound **3**; Figure S10: ESI^+^—MS/MS spectra of compound **4**; Figure S11: Fragmentation pattern for compound **4**; Figure S12: ESI^+^—MS/MS spectra of compound **5**; Figure S13: Fragmentation pattern for compound **5**.

## References

[1] Byregowda R., Prasad S.R., Oelmüller R., Nataraja K.N., Kumar M.K.P. (2022). Is Endophytic Colonization of Host Plants a Method of Alleviating Drought Stress? Conceptualizing the Hidden World of Endophytes. Int. J. Mol. Sci..

[2] Manganyi M.C., Ateba C.N. (2020). Untapped Potentials of Endophytic Fungi: A Review of Novel Bioactive Compounds with Biological Applications. Microorganisms.

[3] Torres-Mendoza D., Ortega H.E., Cubilla-Rios L. (2020). Patents on Endophytic Fungi Related to Secondary Metabolites and Biotransformation Applications. J. Fungi.

[4] Ortega H.E., Torres-Mendoza D., Cubilla-Rios L. (2020). Patents on Endophytic Fungi for Agriculture and Bio- and Phytoremediation Applications. Microorganisms.

[5] Bogas A.C., Cruz F.P.N., Lacava P.T., Sousa C.P. (2022). Endophytic Fungi: An Overview on Biotechnological and Agronomic Potential Fungos Endofíticos: Uma Visão Geral Sobre o Potencial Biotecnológico e Agronômico. Braz. J. Biol..

[6] Demers D.H., Knestrick M.A., Fleeman R., Tawfik R., Azhari A., Souza A., Vesely B., Netherton M., Gupta R., Colon B.L. (2018). Exploitation of Mangrove Endophytic Fungi for Infectious Disease Drug Discovery. Mar. Drugs.

[7] Ancheeva E., Daletos G., Proksch P. (2018). Lead Compounds from Mangrove-Associated Microorganisms. Mar. Drugs.

[8] Cadamuro R.D., Maria I., Bastos S., Silva I.T., Cristiane A., Robl D., Sandjo L.P., Alves S., Lorenzo J.M., Rodr D. (2021). Bioactive Compounds from Mangrove Endophytic Fungus and Their Uses for Microorganism Control. J. Fungi.

[9] Deshmukh S.K., Gupta M.K., Prakash V., Reddy M.S. (2018). Mangrove-Associated Fungi: A Novel Source of Potential Anticancer Compounds. J. Fungi.

[10] Ortega H.E., Torres-Mendoza D., E Z.C., Cubilla-Rios L. (2021). Structurally Uncommon Secondary Metabolites Derived from Endophytic Fungi. J. Fungi.

[11] Salvatore M.M., Alves A., Andolfi A. (2020). Secondary Metabolites of *Lasiodiplodia theobromae*: Distribution, Chemical Diversity, Bioactivity, and Implications of Their Occurrence. Toxins.

[12] Philippini R.R., Martiniano S.E., Franco Marcelino P.R., Chandel A.K., dos Santos J.C., Da Silva S.S. (2021). Production of β-Glucan Exopolysaccharide Lasiodiplodan by *Lasiodiplodia theobromae* CCT 3966 from Corn Bran Acid Hydrolysate. Appl. Microbiol. Biotechnol..

[13] Félix C., Libório S., Nunes M., Félix R., Duarte A.S., Alves A., Esteves A.C. (2018). *Lasiodiplodia theobromae* as a Producer of Biotechnologically Relevant Enzymes. Int. J. Mol. Sci..

[14] Cabezas Gómez O., Barbosa Moreira D.M., Hortolan Luiz J.H. (2021). Medicinal Potentialities and Pathogenic Profile of *Lasiodiplodia* Genus. World J. Microbiol. Biotechnol..

[15] Kumar S., Pagar A.D., Ahmad F., Dwibedi V., Wani A., Bharatam P.V., Chhibber M., Saxena S., Pal Singh I. (2019). Xanthine Oxidase Inhibitors from an Endophytic Fungus *Lasiodiplodia pseudotheobromae*. Bioorg Chem..

[16] Pellissier L., Koval A., Marcourt L., Ferreira Queiroz E., Lecoultre N., Leoni S., Quiros-Guerrero L.M., Barthélémy M., Duivelshof B.L., Guillarme D. (2021). Isolation and Identification of Isocoumarin Derivatives with Specific Inhibitory Activity Against Wnt Pathway and Metabolome Characterization of *Lasiodiplodia venezuelensis*. Front. Chem..

[17] Pellissier L., Leoni S., Marcourt L., Queiroz E.F., Lecoultre N., Quiros-Guerrero L.M., Barthélémy M., Eparvier V., Chave J., Stien D. (2021). Characterization of *Pseudomonas aeruginosa* Quorum Sensing Inhibitors from the Endophyte *Lasiodiplodia venezuelensis* and Evaluation of Their Antivirulence Effects by Metabolomics. Microorganisms.

[18] Li J., Xue Y., Yuan J., Lu Y., Zhu X., Lin Y., Liu L. (2016). Lasiodiplodins from Mangrove Endophytic Fungus *Lasiodiplodia* sp. 318#. Nat. Prod. Res..

[19] Huang J., Xu J., Wang Z., Khan D., Niaz S.I., Zhu Y., Lin Y., Li J., Liu L. (2017). New Lasiodiplodins from Mangrove Endophytic Fungus *Lasiodiplodia* sp. 318#. Nat. Prod. Res..

[20] Chen S., Liu Z., Liu H., Long Y., Chen D., Lu Y., She Z. (2017). Lasiodiplactone A, a Novel Lactone from the Mangrove Endophytic Fungus *Lasiodiplodia theobromae* ZJ-HQ1. Org. Biomol. Chem..

[21] Sato S., Sofian F.F., Suehiro W., Harneti D., Maharani R., Supratman U., Abdullah F.F., Salam S., Koseki T., Shiono Y. (2021). β-Resorcylic Acid Derivatives, with Their Phytotoxic Activities, from the Endophytic Fungus *Lasiodiplodia theobromae* in the Mangrove Plant *Xylocarpus granatum*. Chem. Biodivers..

[22] Chen S., Chen D., Cai R., Cui H., Long Y., Lu Y., Li C., She Z. (2016). Cytotoxic and Antibacterial Preussomerins from the Mangrove Endophytic Fungus *Lasiodiplodia theobromae* ZJ-HQ1. J. Nat. Prod..

[23] Matsui R., Amano N., Takahashi K., Taguchi Y., Saburi W., Mori H., Kondo N., Matsuda K., Matsuura H. (2017). Elucidation of the Biosynthetic Pathway of Cis-Jasmone in *Lasiodiplodia theobromae*. Sci. Rep..

[24] Eng F., Marin J.E., Zienkiewicz K., Gutiérrez-Rojas M., Favela-Torres E., Feussner I. (2021). Jasmonic Acid Biosynthesis by Fungi: Derivatives, First Evidence on Biochemical Pathways and Culture Conditions for Production. PeerJ.

[25] Eng F., Haroth S., Feussner K., Meldau D., Rekhter D., Ischebeck T., Brodhun F., Feussner I. (2016). Optimized Jasmonic Acid Production by *Lasiodiplodia theobromae* Reveals Formation of Valuable Plant Secondary Metabolites. PLoS ONE.

[26] Andolfi A., Maddau L., Cimmino A., Linaldeddu B.T., Basso S., Deidda A., Serra S., Evidente A. (2014). Lasiojasmonates A-C, Three Jasmonic Acid Esters Produced by *Lasiodiplodia* sp., a Grapevine Pathogen. Phytochemistry.

[27] Shen Z., Zheng P., Li R., Sun X., Chen P., Wu D. (2022). High Production of Jasmonic Acid by *Lasiodiplodia iranensis* Using Solid-State Fermentation: Optimization and Understanding. Biotechnol. J..

[28] Shyaula S.L., Singh Y., Rawat P., Kanojiya S. (2023). Comprehensive Characterization of Dactylorhin and Loroglossin in *Dactylorhiza hatagirea*, D. DON Using Ultra-Performance Liquid Chromatography-Mass Spectrometry. Int. J. Mass Spectrom..

[29] Liu Z.Y., Yu C.H., Wan L., Sun Z.L. (2012). Fragmentation Study of Five Trichothecenes Using Electrospray Hybrid Ion Trap/Time-of-Flight Mass Spectrometry with Accurate Mass Measurements. Int. J. Mass Spectrom..

[30] Lijina P., Gnanesh Kumar B.S. (2023). Differentiating Planteose and Raffinose Using Negative Ion Mode Mass Spectrometry. Int. J. Mass Spectrom..

[31] Molinar E., Rios N., Spadafora C., Elizabeth Arnold A., Coley P.D., Kursar T.A., Gerwick W.H., Cubilla-Rios L. (2012). Coibanoles, a New Class of Meroterpenoids Produced by *Pycnoporus sanguineus*. Tetrahedron Lett..

[32] Humber R.A., Lacey L.A. (1997). Fungi: Preservation of Cultures. Manual of Techniques in Insect Pathology.

[33] Harris J.L. (2000). Letter to the Editor Safe, Low-Distortion Tape Touch Method for Fungal Slide Mounts. J. Clin. Microbiol..

[34] Raja H.A., Miller A.N., Pearce C.J., Oberlies N.H. (2017). Fungal Identification Using Molecular Tools: A Primer for the Natural Products Research Community. J. Nat. Prod..

[35] White T.J., Bruns T., Lee S., Taylor J. (1990). Amplification and direct sequencing of fungal ribosomal RNA genes for phylogenetics. PCR Protocols.

[36] Hall T.A. (1999). BioEdit: A user_friendly biological sequence Alignment Editor and Analysis Program for Windows 95/98 NT. Nucleic Acids Symp. Ser..

[37] Hall T.A. (1991). BioEdit: An Important Software for Molecular Biology. GERF Bull. Biosci..

[38] Altschup S.F., Gish W., Miller W., Myers E.W., Lipman D.J. (1990). Basic Local Alignment Search Tool. J. Mol. Biol..

[39] Abdollahzadeh J., Javadi A., Goltapeh E.M., Zare R., Phillips A.J.L. (2010). Phylogeny and Morphology of Four New Species of *Lasiodiplodia* from Iran. Persoonia Mol. Phylogeny Evol. Fungi.

[40] Rodríguez-Gálvez E., Guerrero P., Barradas C., Crous P.W., Alves A. (2017). Phylogeny and Pathogenicity of *Lasiodiplodia* Species Associated with Dieback of Mango in Peru. Fungal Biol..

[41] Wang Y., Lin S., Zhao L., Sun X., He W., Zhang Y., Dai Y.-C. (2019). *Lasiodiplodia* spp. Associated with *Aquilaria crassna* in Laos. Mycol. Prog..

[42] Tamura K., Stecher G., Kumar S. (2021). MEGA11: Molecular Evolutionary Genetics Analysis Version 11. Mol. Biol. Evol..

[43] Hasegawa M., Kishino H., Yano T.A. (1985). Dating of the Human-Ape Splitting by a Molecular Clock of Mitochondrial DNA. J. Mol. Evol..

[44] Martínez-Luis S., Ballesteros J., Gutiérrez M. (2011). Antibacterial Constituents from the Octocoral-Associated Bacterium *Pseudoalteromonas* sp.. Rev. Latinoamer. Quím.

[45] Castillo G., Torrecillas A., Nogueiras C., Michelena G., Sánchez-Bravo J., Acosta M. (2014). Simultaneous Quantification of Phytohormones in Fermentation Extracts of *Botryodiplodia theobromae* by Liquid Chromatography-Electrospray Tandem Mass Spectrometry. World J. Microbiol. Biotechnol..

[46] El-Ganainy S.M., Ismail A.M., Iqbal Z., Elshewy E.S., Alhudaib K.A., Almaghasla M.I., Magistà D. (2022). Diversity among *Lasiodiplodia* Species Causing Dieback, Root Rot and Leaf Spot on Fruit Trees in Egypt, and a Description of *Lasiodiplodia newvalleyensis* sp. nov. J. Fungi.

[47] Reid G.E., Simpson R.J., O’Hair R.A.J. (1998). A Mass Spectrometric and Ab Initio Study of the Pathways for Dehydration of Simple Glycine and Cysteine-Containing Peptide [M+H]+ Ions. J. Am. Soc. Mass. Spectrom..

[48] Beynon J.G., Caprioli R.M., Shannon T.W. (1971). The Structure of the Product Ion of a ‘McLafferty’ Rearrangement. Org. Mass. Spectrom..

[49] Petersson G. (1972). A McLafferty-type Rearrangement of a Trimethylsilyl Group in Silylated Hydroxy Carbonyl Compounds. Org. Mass. Spectrom..

[50] Demarque D.P., Crotti A.E.M., Vessecchi R., Lopes J.L.C., Lopes N.P. (2016). Fragmentation Reactions Using Electrospray Ionization Mass Spectrometry: An Important Tool for the Structural Elucidation and Characterization of Synthetic and Natural Products. Nat. Prod. Rep..

[51] Gross M.L. (1992). Charge-Remote Fragmentations: Method, Mechanism and Applications. Int. J. Mass Spectrom. Ion. Process.

[52] Furtado N.A.J.C., Vessecchi R., Tomaz J.C., Galembeck S.E., Bastos J.K., Lopes N.P., Crotti A.E.M. (2007). Fragmentation of Diketopiperazines from *Aspergillus fumigatus* by Electrospray Ionization Tandem Mass Spectrometry (ESI-MS/MS). J. Mass Spectrom..

[53] Oh H.B., Moon B. (2015). Radical-Driven Peptide Backbone Dissociation Tandem Mass Spectrometry. Mass Spectrom. Rev..

[54] Kokuev A.O., Sukhorukov A.Y. (2023). Michael Addition of *P*-Nucleophiles to Azoalkenes Provides Simple Access to Phosphine Oxides Bearing an Alkylhydrazone Moiety. Front. Chem..

[55] Vessecchi R., Julião Zocolo G., Rubio Gouvea D., Hübner F., Cramer B., Rodrigues De Marchi M.R., Humpf H.U., Lopes N.P. (2011). Re-Examination of the Anion Derivatives of Isoflavones by Radical Fragmentation in Negative Electrospray Ionization Tandem Mass Spectrometry: Experimental and Computational Studies. Rapid Commun. Mass. Spectrom..

[56] Ueda M., Kaji T., Kozaki W. (2020). Recent Advances in Plant Chemical Biology of Jasmonates. Int. J. Mol. Sci..

[57] Li Z.G., Chen K.X., Xie H.Y., Gao J.R. (2009). Quantitative Structure-Property Relationship Studies on Amino Acid Conjugates of Jasmonic Acid as Defense Signaling Molecules. J. Integr. Plant Biol..

