# Supplementary material for: Identification of Secondary Metabolites from the Mangrove-Endophyte Lasiodiplodia iranensis F0619 by UPLC-ESI-MS/MS
Source: Metabolites. 2023 Aug 3;13(8):912. doi: 10.3390/metabo13080912 (PMC10456654; doi:10.3390/metabo13080912)
Supplement: Supplementary file 1 [file metabolites-13-00912-s001.zip › metabolites-2496938-supplementary.pdf]

Supporting Information

Identification of Secondary Metabolites from the Mangrove-Endophyte *Lasiodiplodia iranensis* F0619 by UPLC-ESI-MS/MS

Lizbeth M. Delgado Gómez [1], Daniel Torres-Mendoza [1,2,3], Kathleen Hernández-Torres [1,4], Humberto E. Ortega [1,2] and Luis Cubilla-Rios [1,2]*

[1] Laboratorio de Bioorgánica Tropical, Facultad de Ciencias Naturales, Exactas y Tecnología, Universidad de Panamá, Panamá 0824, Panama; 13liz2014@gmail.com (L.M.D.G.); daniel-t.torres-m@up.ac.pa (D.T.-M.); kathleen-j.hernandez-t@up.ac.pa (K.H.-T.); humberto.ortegad@up.ac.pa (H.E.O.)

[2] Departamento de Química Orgánica, Facultad de Ciencias Naturales, Exactas y Tecnología, Universidad de Panamá, Panamá 0824, Panama

[3] Vicerrectoría de Investigación y Postgrado, Universidad de Panamá, Panamá 0824, Panama

[4] Programa de Maestría en Microbiología Ambiental, Vicerrectoría de Investigación y Postgrado, Universidad de Panamá, Panamá 0824, Panama

* Correspondence: luis.cubilla@up.ac.pa; Tel.: +507-6676-5824

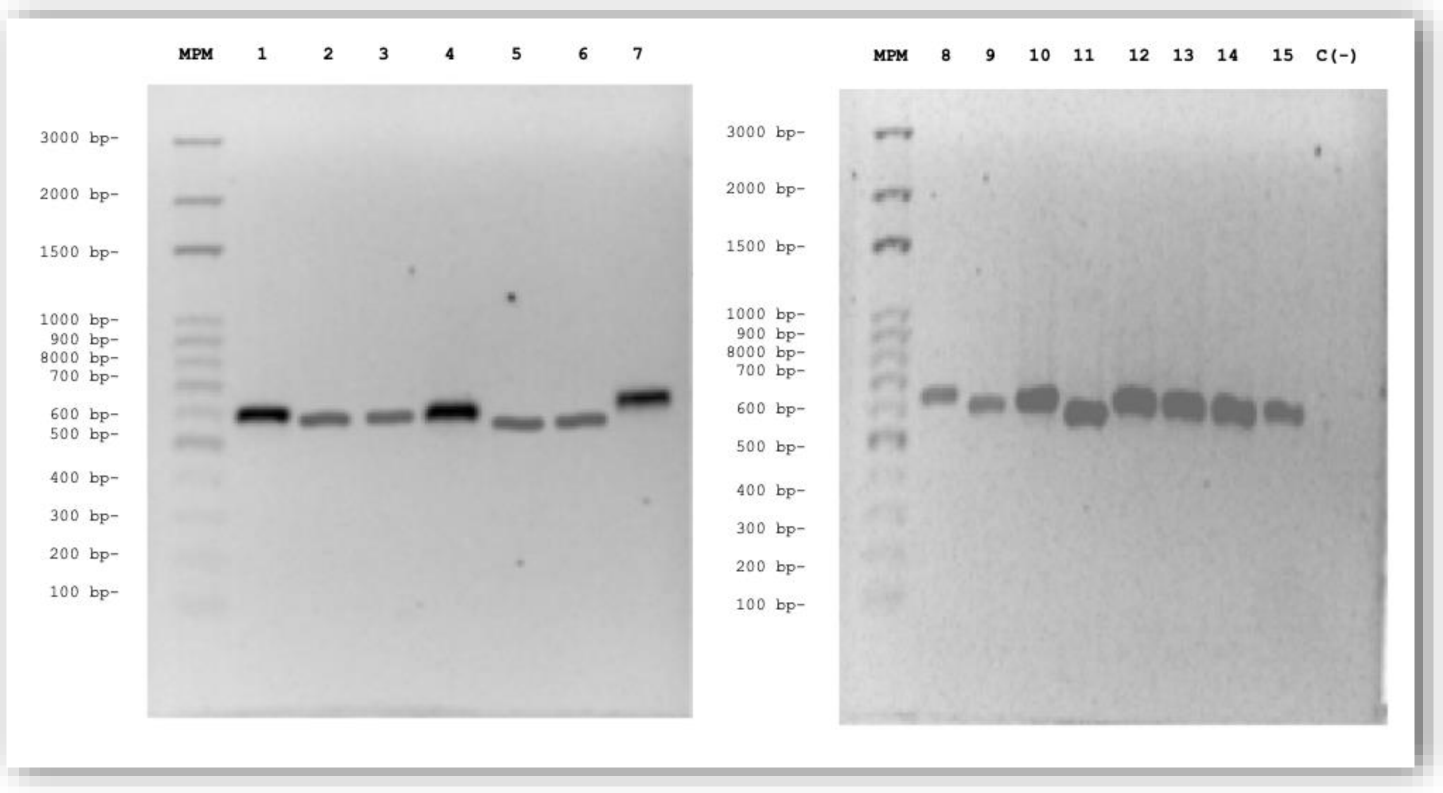

Figure S1. The quality of PCR products was checked on 0.9% agarose electrophoresis gels. Here, lane 1 represent the ITS amplicon of *Lasiodiplodia iranensis* F0619 (600 bp), and lanes 2-15 represents samples for ITS amplicon for different fungi strains. Lane C(-) represent PCR negative control. MPM is the 100 bp molecular weight marker.

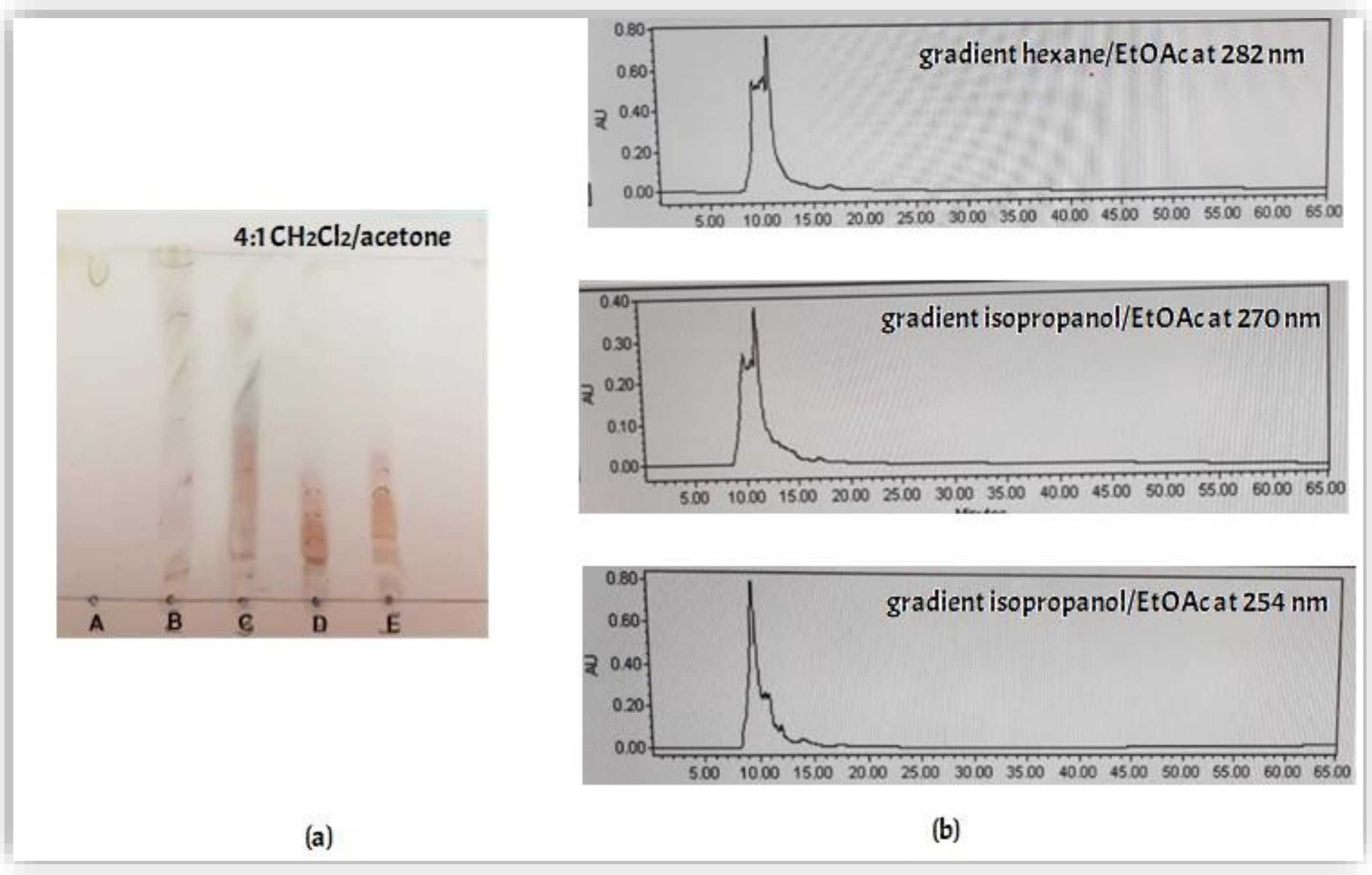

Figure S2. TLC and HPLC analysis of fractions of *Lasiodiplodia iranensis* F0619. **(a)** Thin Layer Chromatography of fractions A-E from the fractionation of crude extract; the spots were eluted using 4:1 CH_2Cl_2/acetone in normal phase silica gel and revealed with *p*-anisaldehyde reagent. **(b)** Chromatograms of HPLC analysis of fraction C with several normal phase silica gel gradients at 1.5 mL/min.

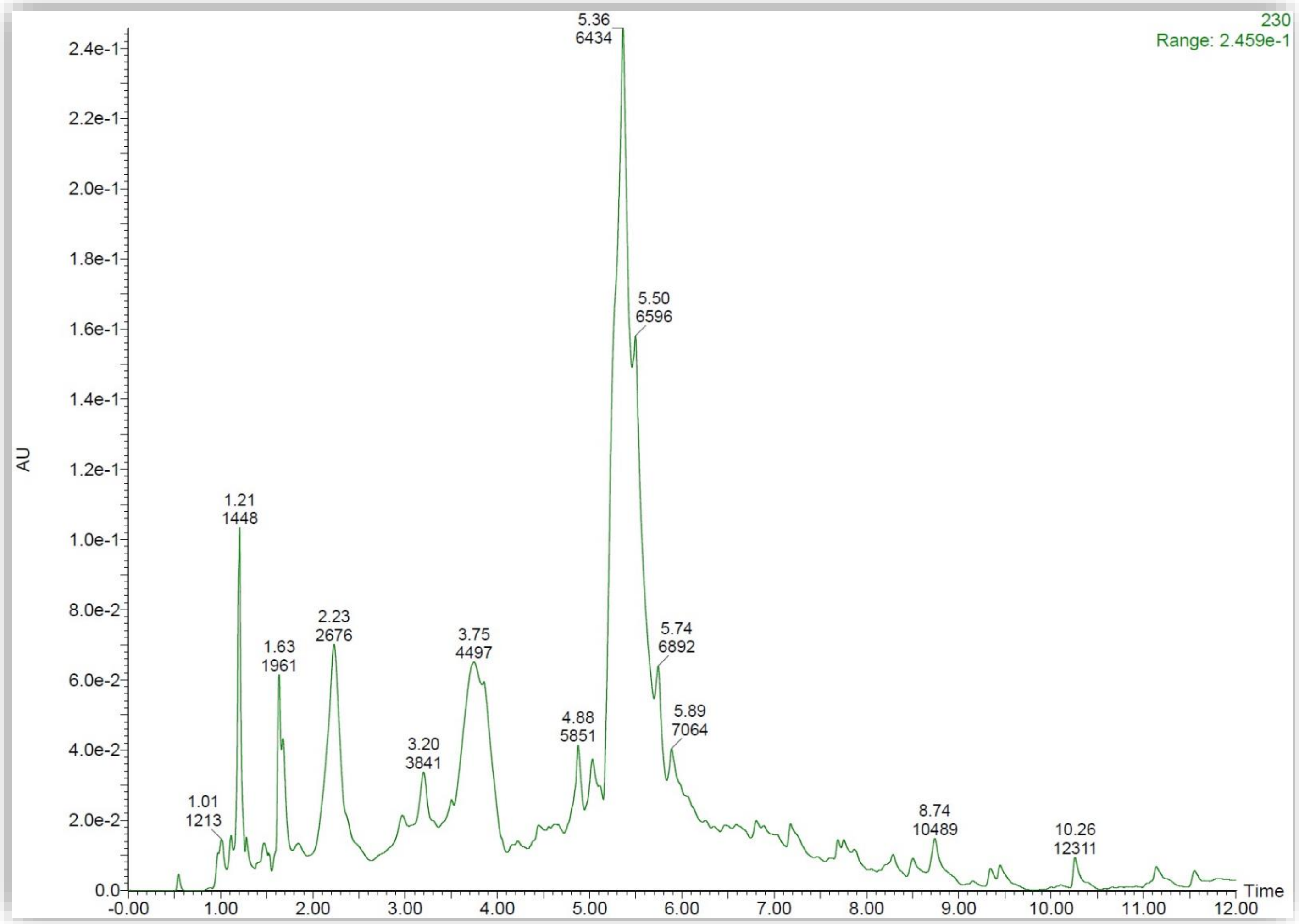

Figure S3. UPLC-DAD chromatogram from fraction C. The AcCN-H2O gradient started from 10:90% AcCN-H_2O to 100% AcCN in 10 minutes and returning to the initial condition in 2 minutes at flow rate of 200 μL/min and monitoring at 230 nm.

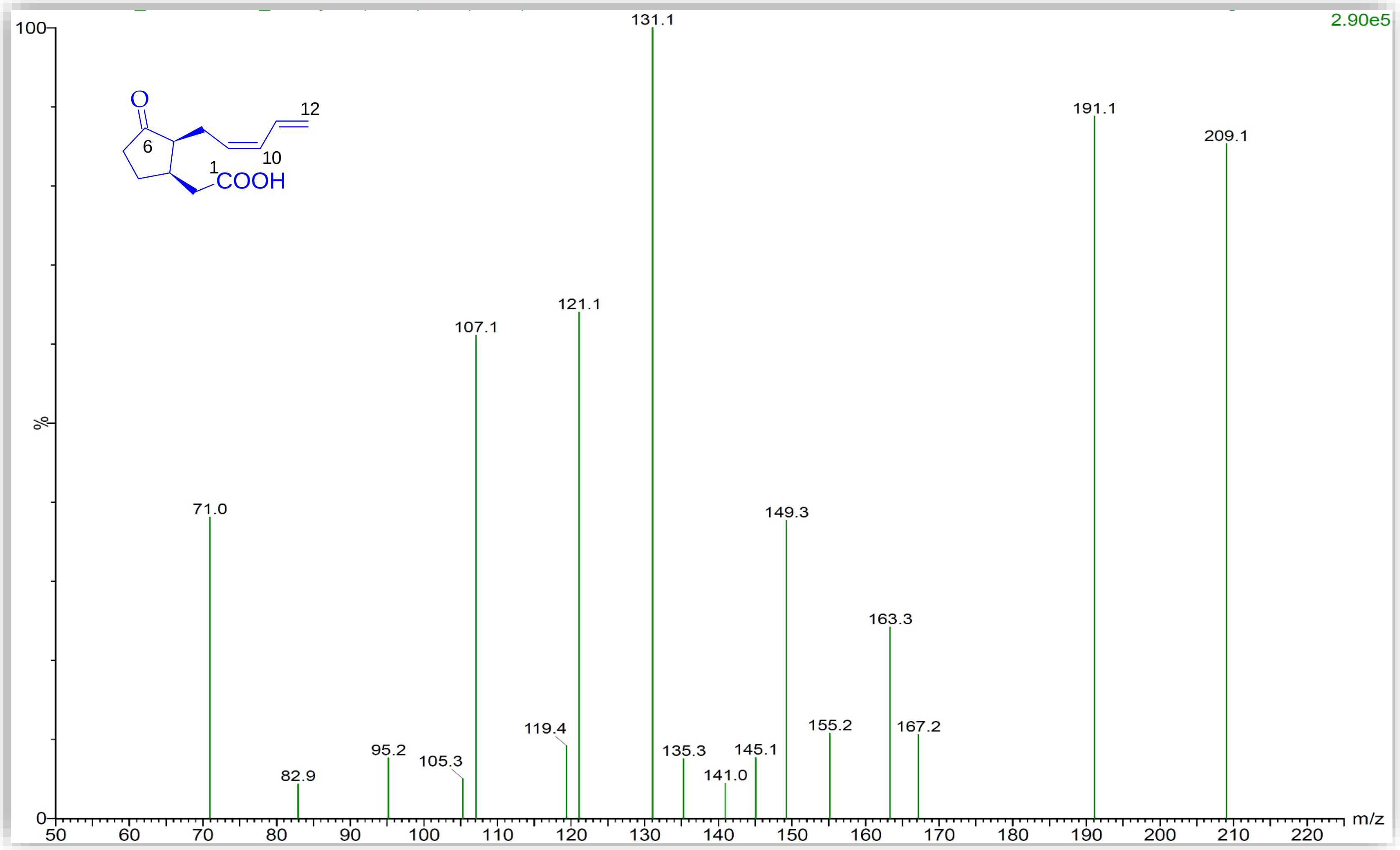

Figure S4. ESI⁺—MS/MS spectra of compound **1**

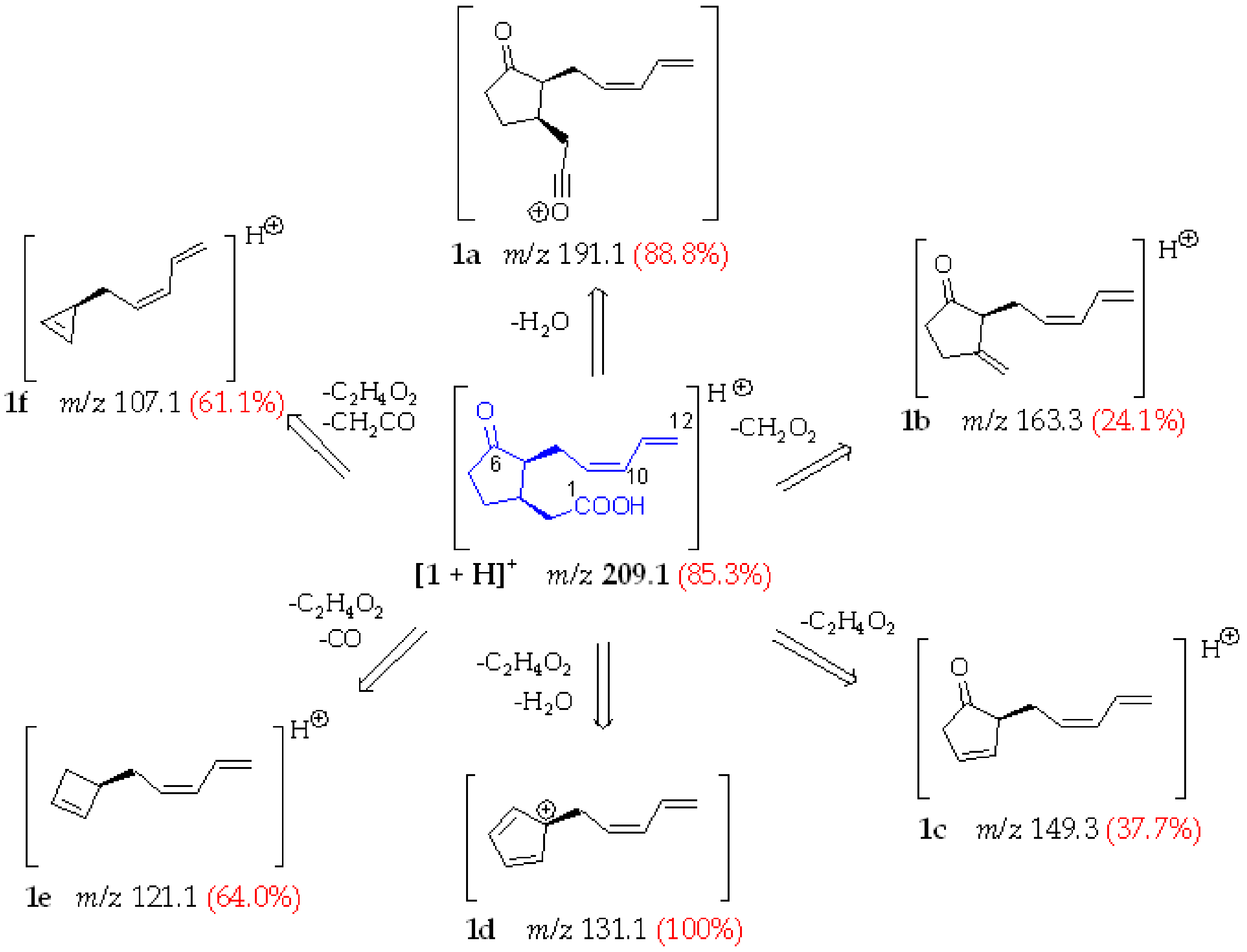

Figure S5. Fragmentation pattern for compound **1**. Relative abundance in parenthesis.

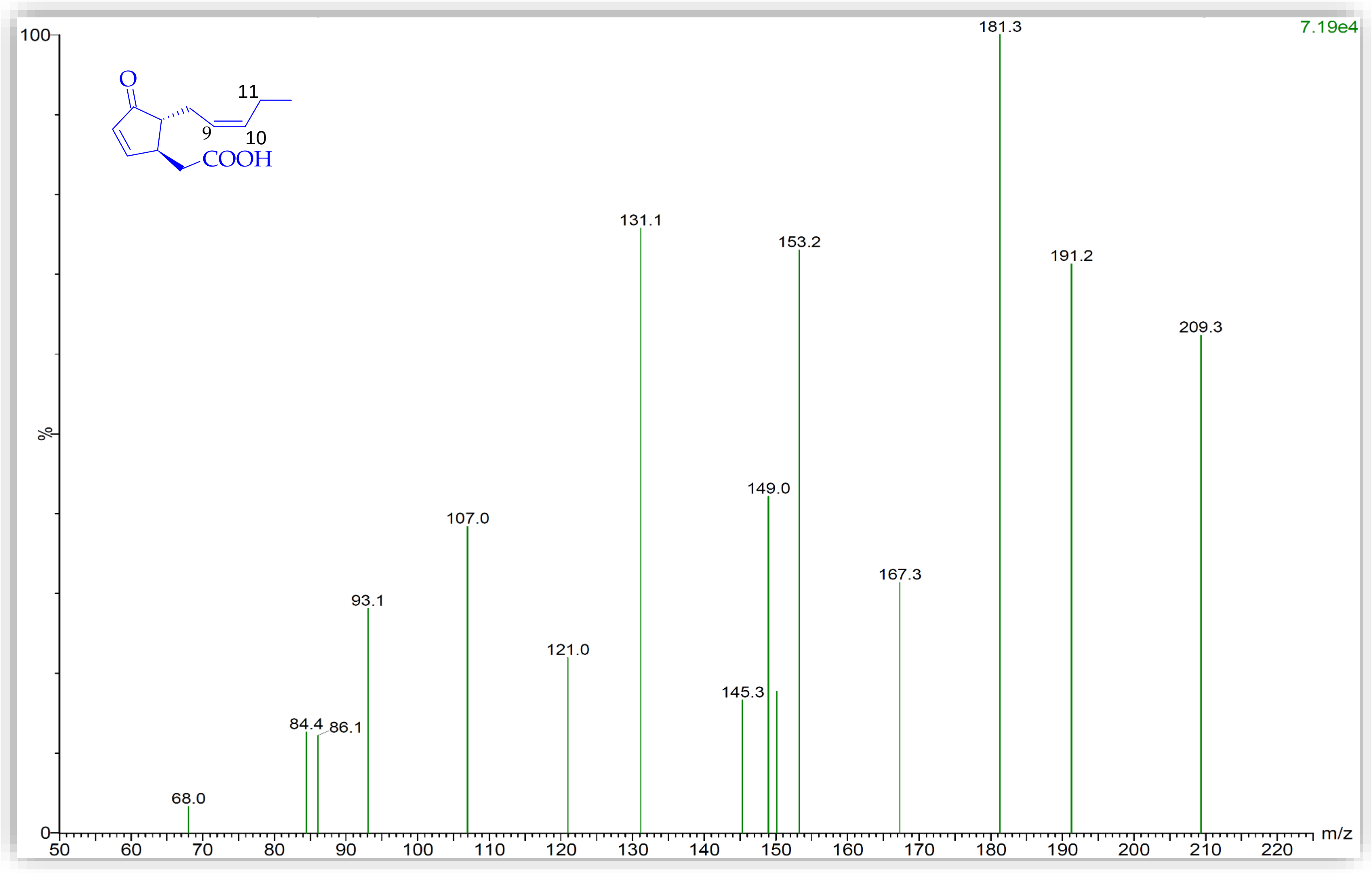

Figure S6. ESI+—MS/MS spectra of compound **2**

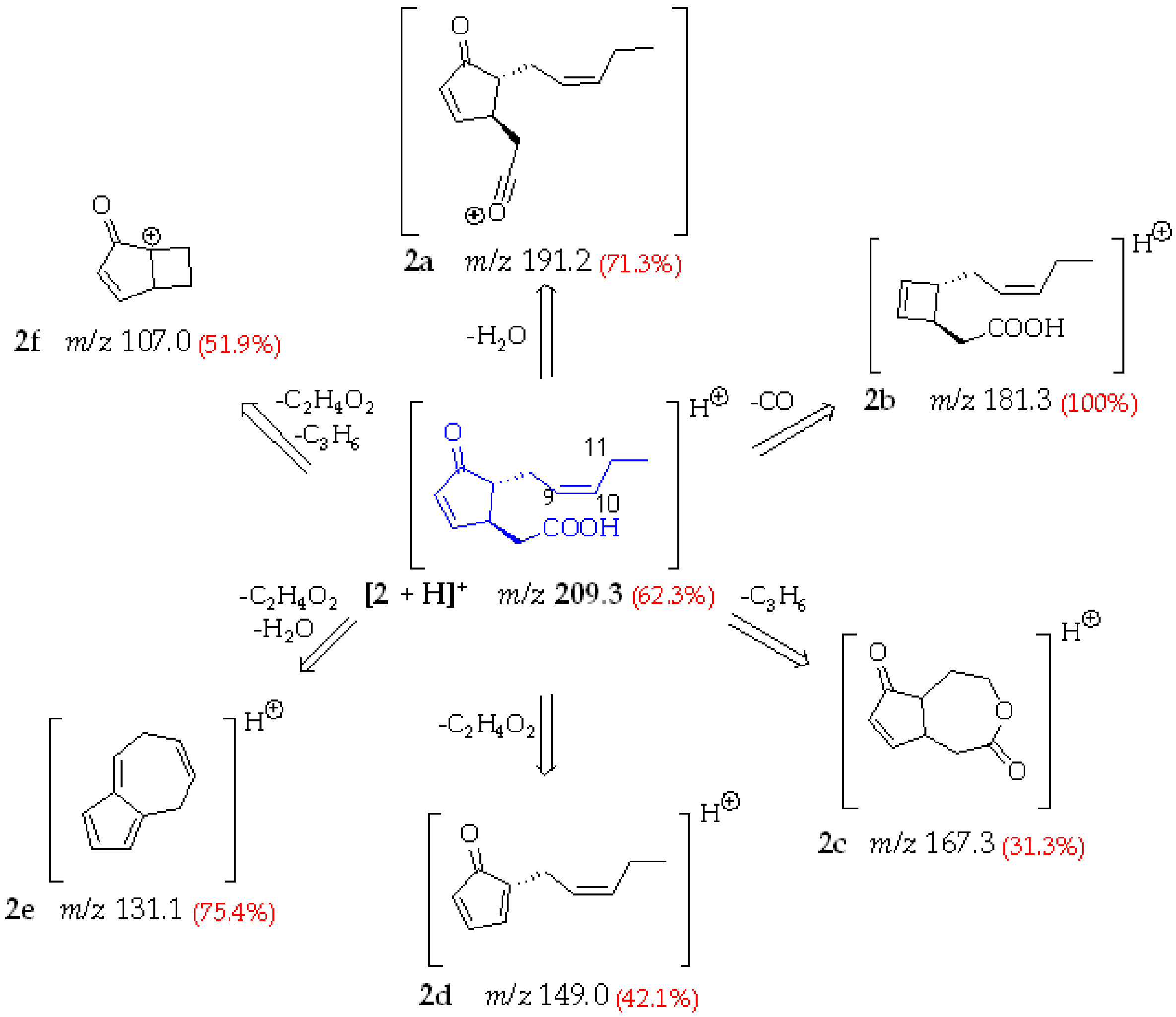

Figure S7. Fragmentation pattern for compound **2**. Relative abundance in parenthesis.

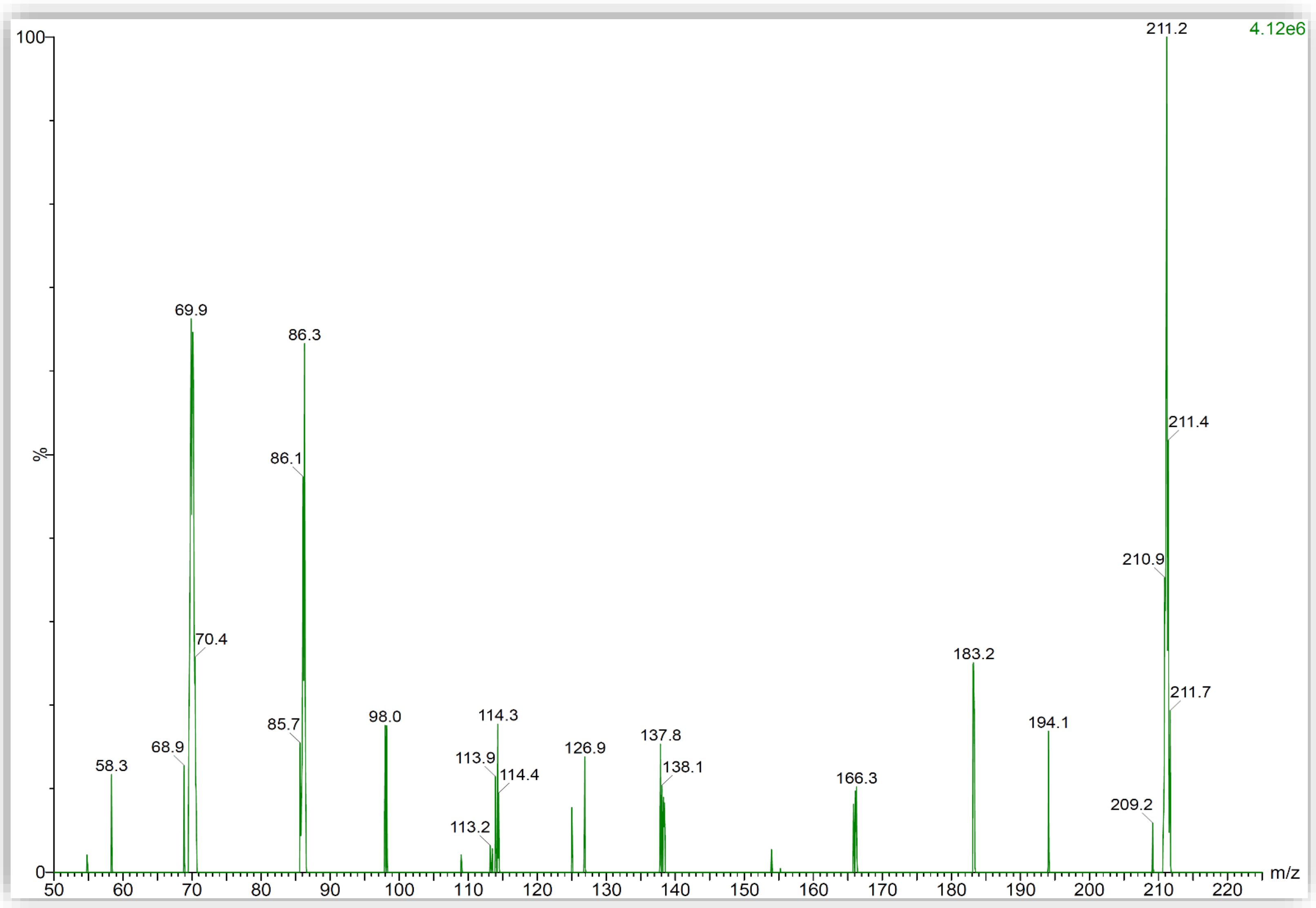

Figure S8. ESI$^+$—MS/MS spectra of compound **3**

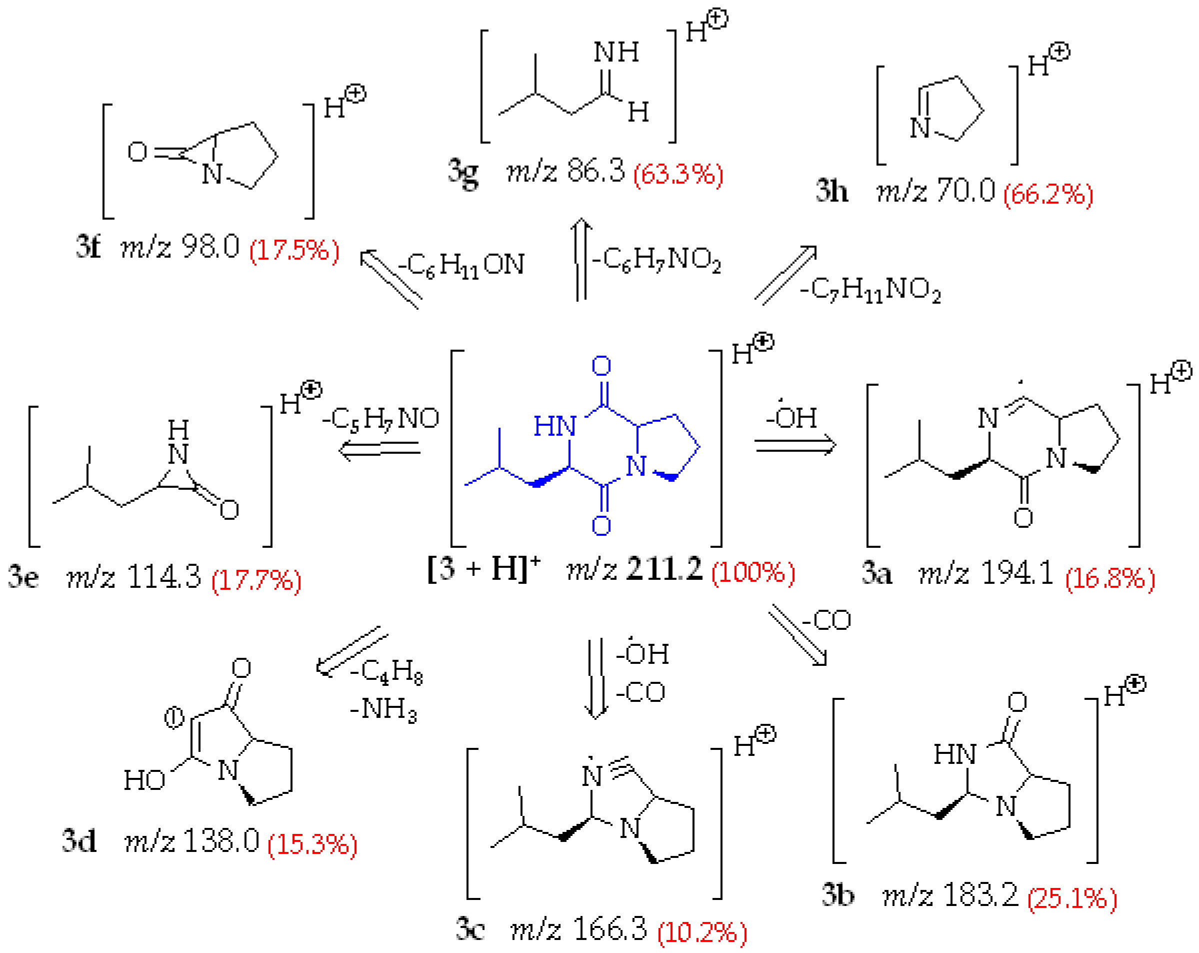

Figure S9. Fragmentation pattern for compound **3**. Relative abundance in parenthesis.

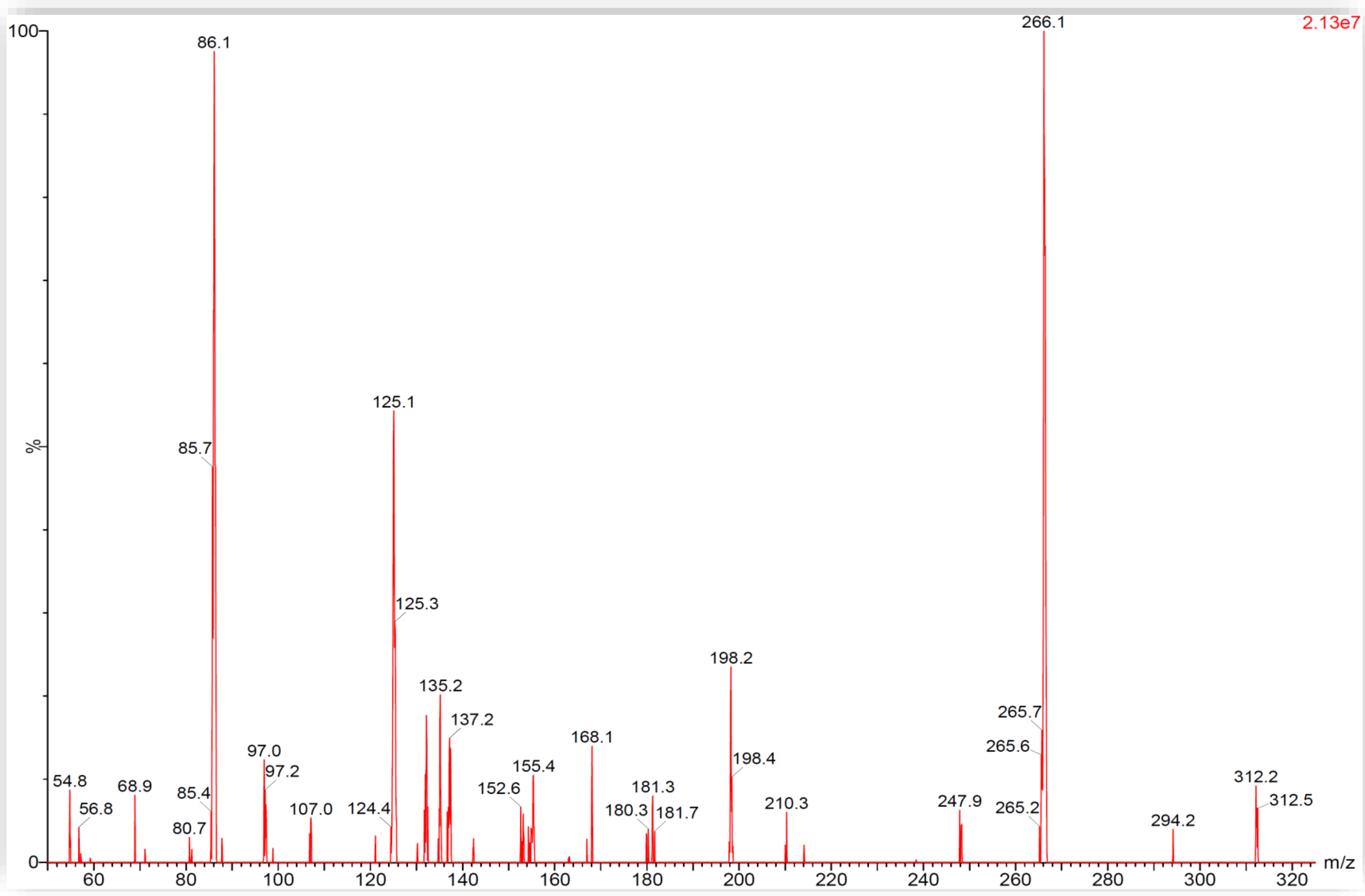

Figure S10. ESI⁺—MS/MS spectra of compound **4**

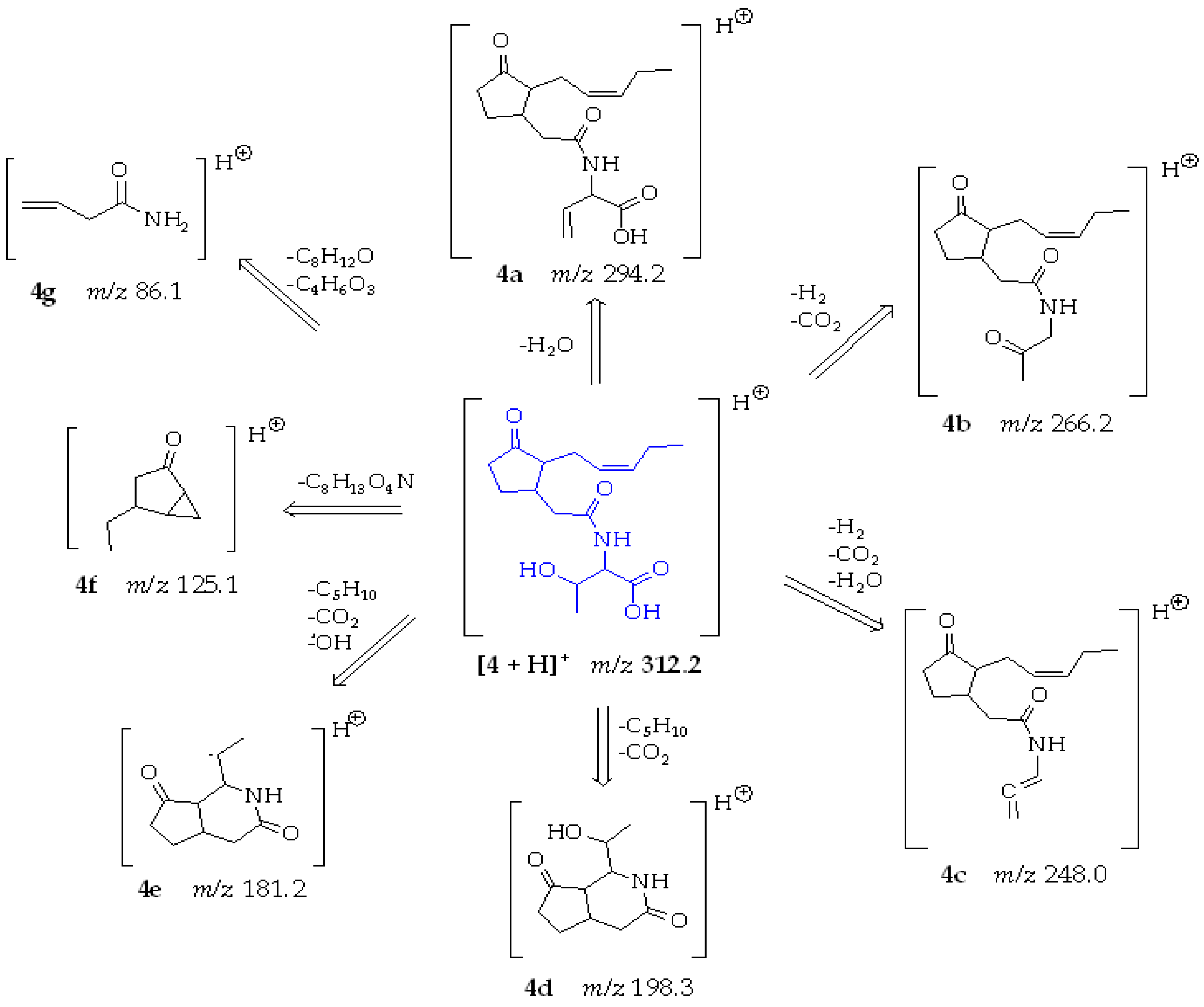

Figure S11. Fragmentation pattern for compound **4**.

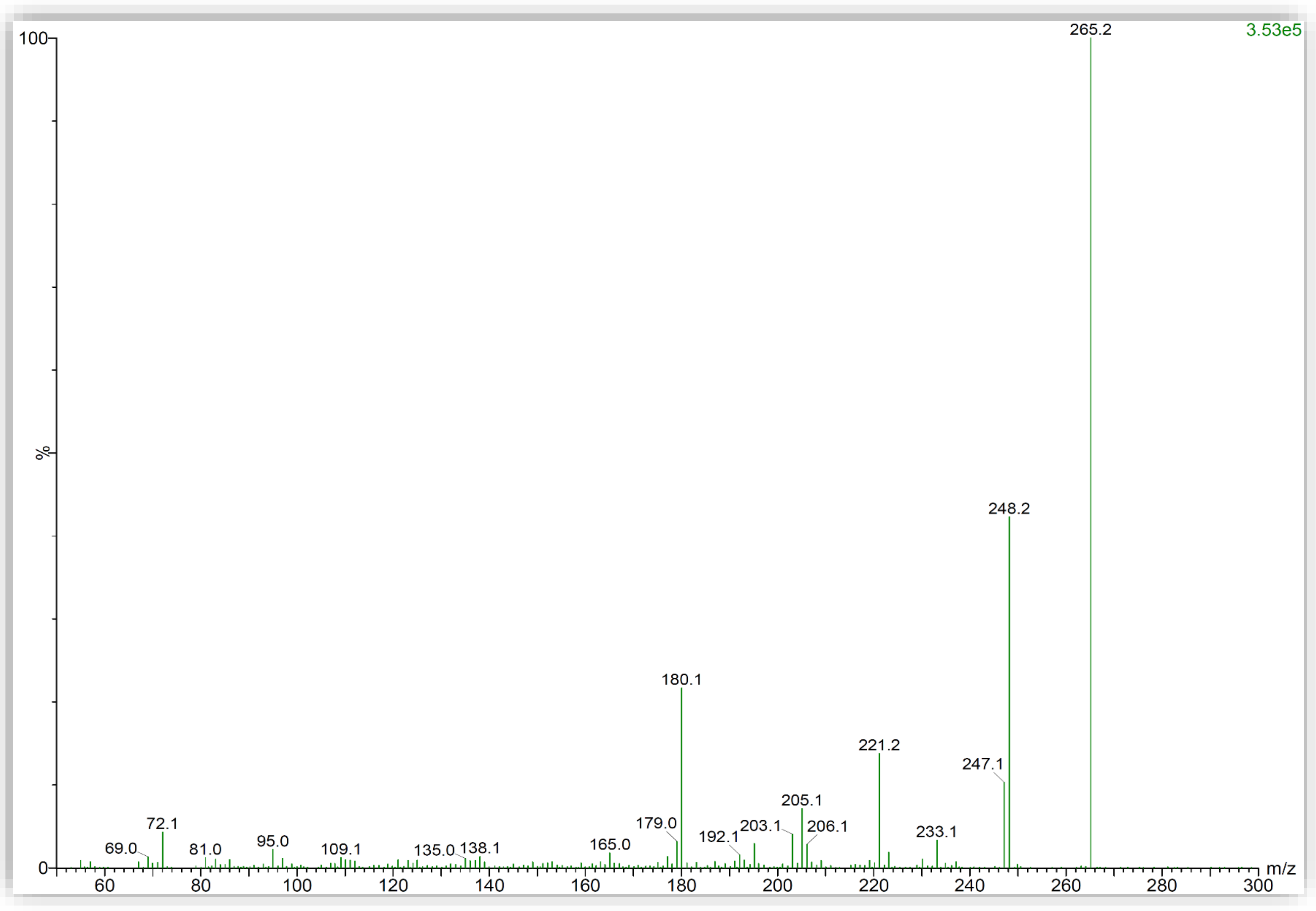

Figure S12. ESI⁺—MS/MS spectra of compound **5**

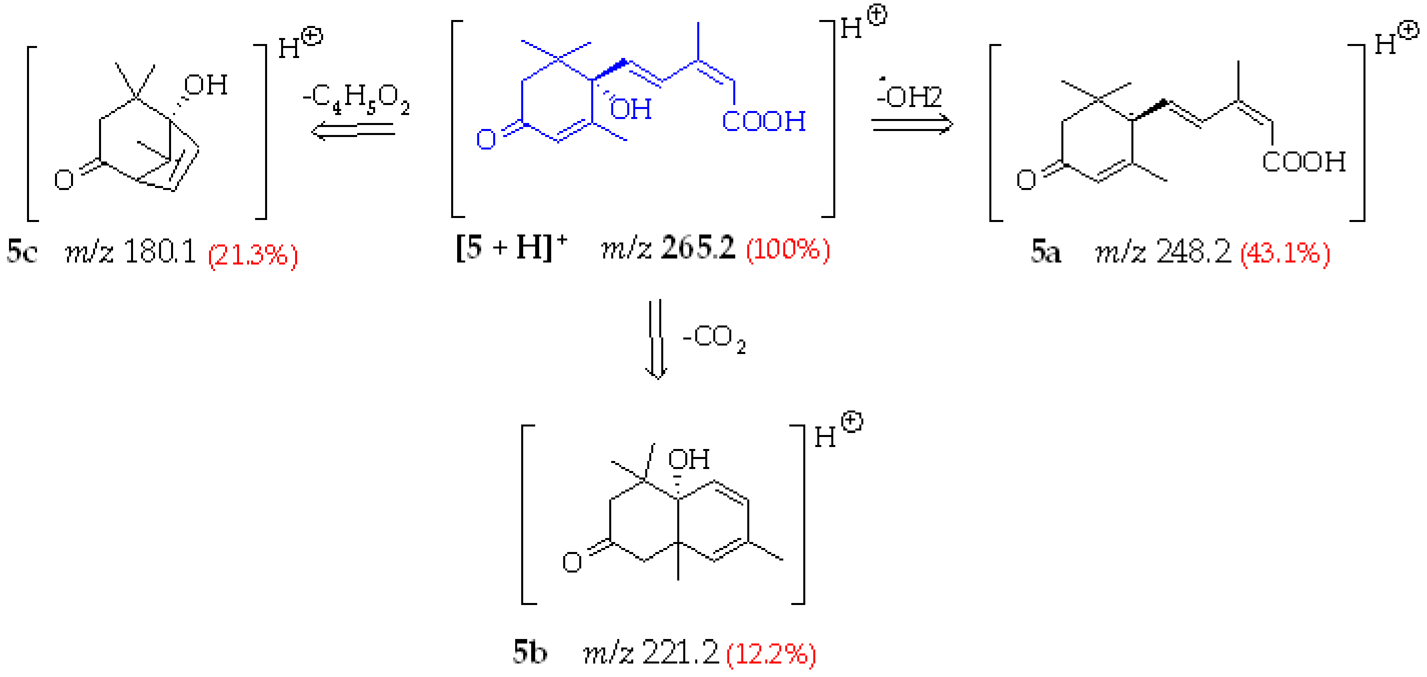

Figure S13. Fragmentation pattern for compound **5**. Relative abundance in parenthesis.
